# Supplementary material for: Structural basis of Cullin 2 RING E3 ligase regulation by the COP9 signalosome
Source: Nat Commun. 2019 Aug 23;10:3814. doi: 10.1038/s41467-019-11772-y (PMC6707232; doi:10.1038/s41467-019-11772-y)
Supplement: Supplementary file 1 — Supplementary Information [file 41467_2019_11772_MOESM1_ESM.pdf]

**Supplementary Information for**

# Structural basis of Cullin 2-RING E3 ligase regulation by the COP9 signalosome

Sarah V. Faull[#], Andy. M. C. Lau[#], Chloe Martens, Zainab Ahdash, Kjetil Hansen, Hugo Yebenes, Carla Schmidt, Fabienne Beuron, Nora B. Cronin, Edward P. Morris[*], Argyris Politis[*]

**#** These authors contributed equally to this work

* Correspondence:

Edward Morris

Tel: +44 (0) 20 7153 5531 Email: ed.morris@icr.ac.uk

Argyris Politis

Tel: +44 (0) 20 7848 7514 Email: argyris.politis@kcl.ac.uk

**This PDF file includes:**

- Supplementary Figures 1 to 26
- Supplementary Table 1
- Supplementary Notes 1 to 2
- Supplementary References

## Supplementary Figures

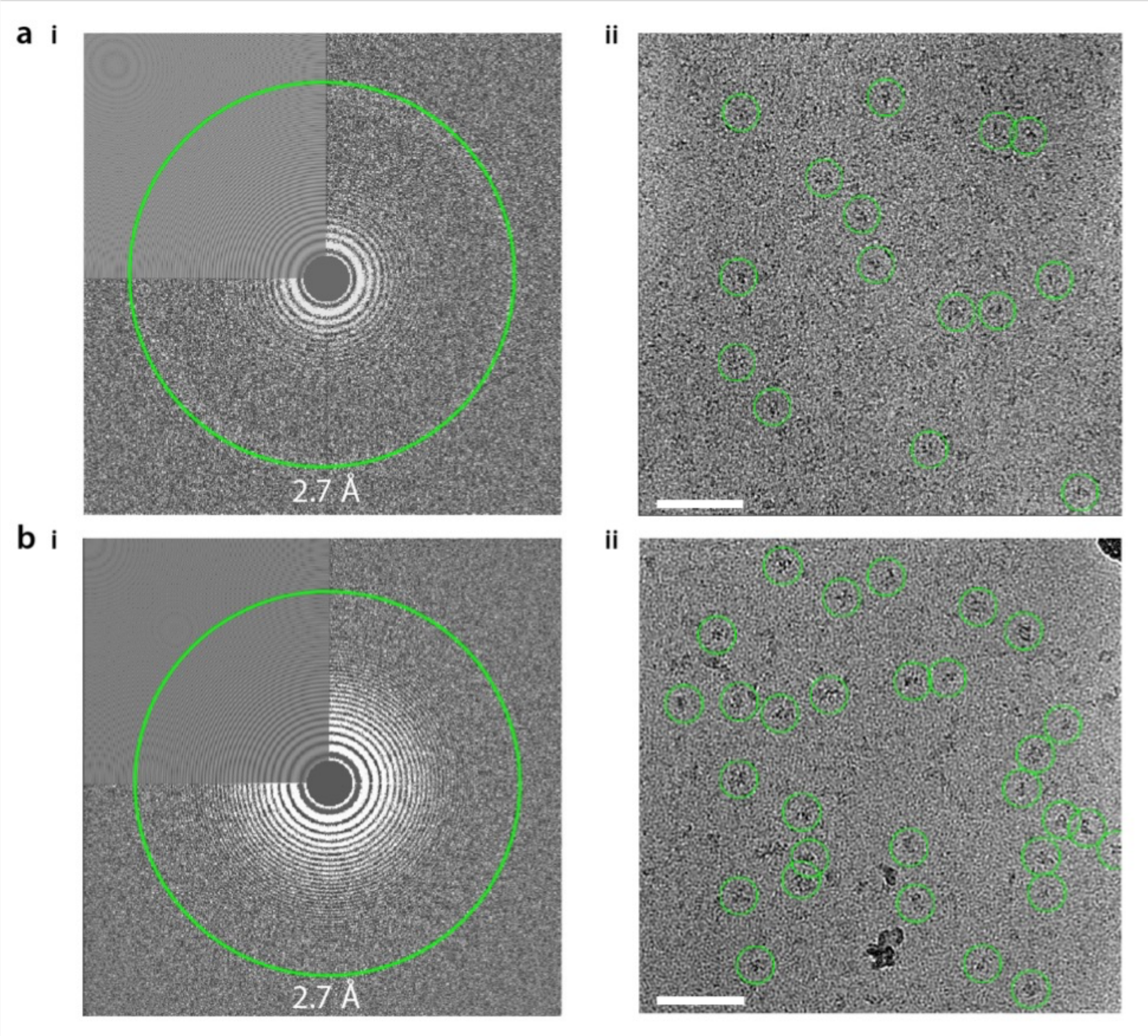


**Supplementary Figure 1. Cryo-electron micrographs.** Micrographs of (**a**) CSN-CRL2~N8 and (**b**) CSN-CRL2 complexes. (**i**) Representative power spectrum and (**ii**) various single molecular views are circled. Scale bar represents 80 nm.

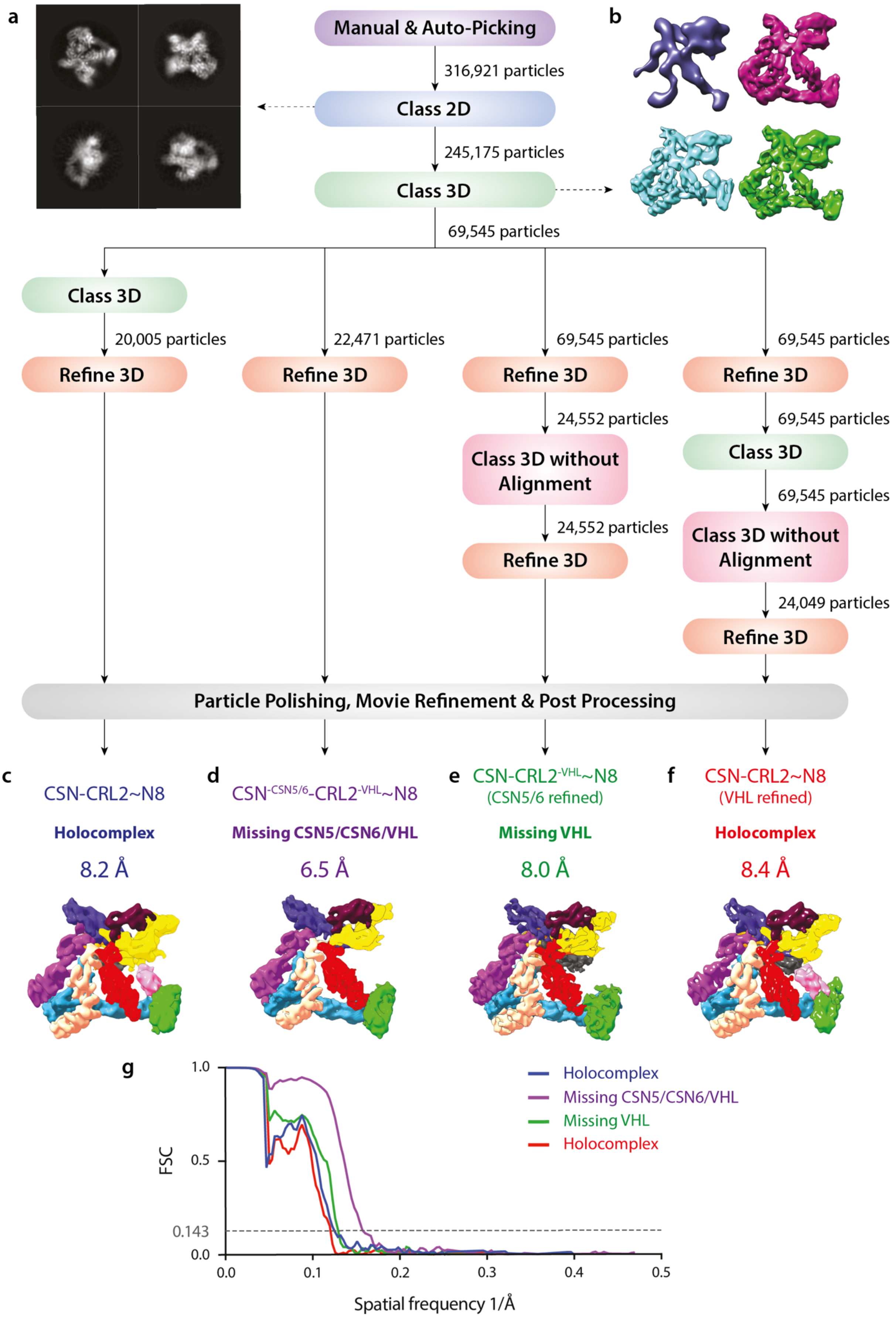

a
b
Manual & Auto-Picking
316,921 particles
Class 2D
245,175 particles
Class 3D
69,545 particles
Class 3D
20,005 particles
Refine 3D
22,471 particles
Refine 3D
69,545 particles
Refine 3D
24,552 particles
Class 3D without Alignment
24,552 particles
Refine 3D
69,545 particles
Refine 3D
69,545 particles
Class 3D
69,545 particles
Class 3D without Alignment
24,049 particles
Refine 3D
Particle Polishing, Movie Refinement & Post Processing
c
CSN-CRL2~N8
Holocomplex
8.2 Å
d
CSN-CSN5/6-CRL2-VHL~N8
Missing CSN5/CSN6/VHL
6.5 Å
e
CSN-CRL2-VHL~N8
(CSN5/6 refined)
Missing VHL
8.0 Å
f
CSN-CRL2~N8
(VHL refined)
Holocomplex
8.4 Å
g
FSC
1.0
0.5
0.143
0.0
0.0
0.1
0.2
0.3
0.4
0.5
Spatial frequency 1/Å
Holocomplex
Missing CSN5/CSN6/VHL
Missing VHL
Holocomplex

**Supplementary Figure 2. Flowchart depicting the workflow for processing cryo-EM data**. A set of ~3100 micrographs were subjected to manual and auto-picking in order to acquire particles for 2D reference-free classification (**a**). 2D classification was used for the positive selection of particles prior to 3D classification. (**b**) Four of the fifteen classes generated, demonstrate subunit heterogeneity and presence of a small component of apo-CSN (purple model) in the data set. Particles from the three models containing the CSN and CRL2~N8 in (**b**) were pooled and further processed as described in the workflow. Maps (**e**) (CSN-CRL2$^{-VHL}$~N8) and (**f**) (CSN-CRL2 ~N8) were generated using focused refinement by applying a mask and using the area of interest as a starting model. (**g**) shows the FSCs for the four maps (**c-f**). Source Data are provided as a Source Data file.

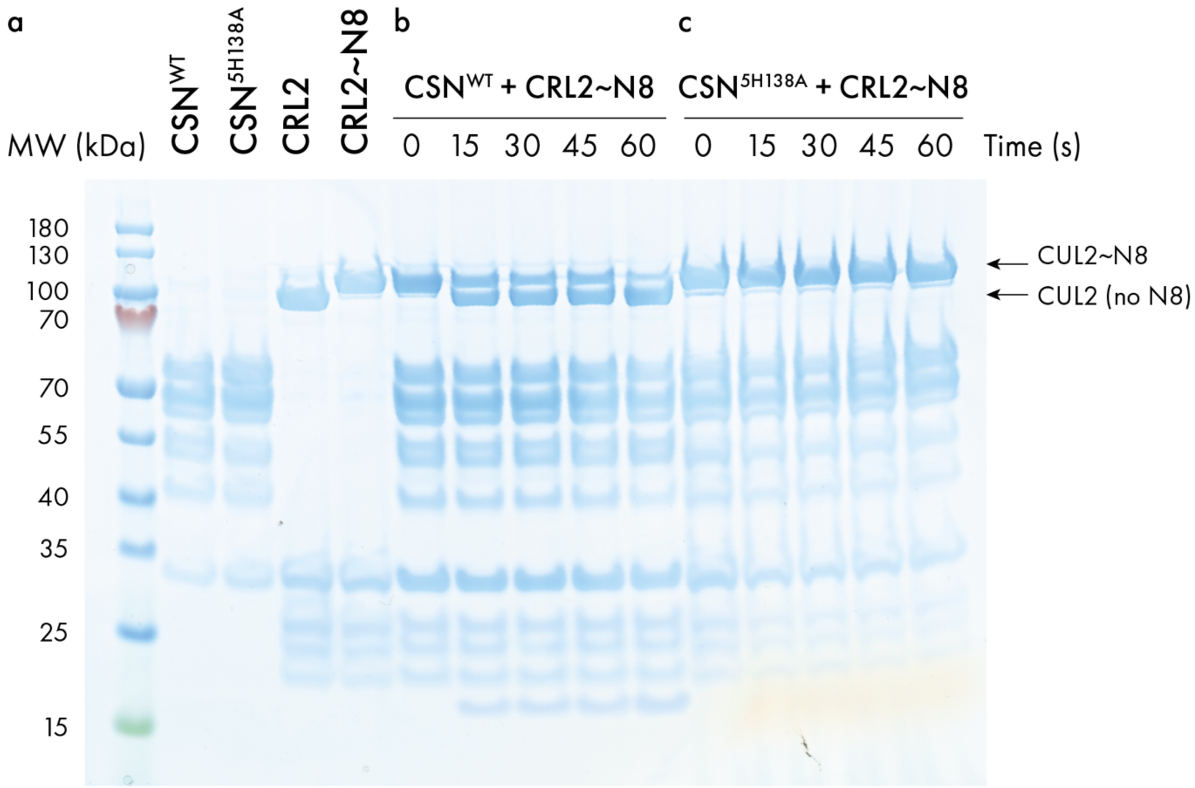


**Supplementary Figure 3. Deneddylation activity of CSN$^{WT}$ and CSN$^{5H138A}$.** (a) Bands corresponding to denatured CSN and CRL2 complexes. (b) Incubation of CSN$^{WT}$ with CRL2~N8 and (c) CSN$^{5H138A}$ with CRL2~N8 over time. Proteins were incubated at 37°C and reactions were inhibited through the addition of lithium dodecyl sulphate (LDS) and quickly heating to 90˚C using a pre-heated heat block to ensure rapid denaturation. Bands corresponding to CUL2~N8 and CUL2 are indicated by arrows for clarity. Source Data are provided as a Source Data file.

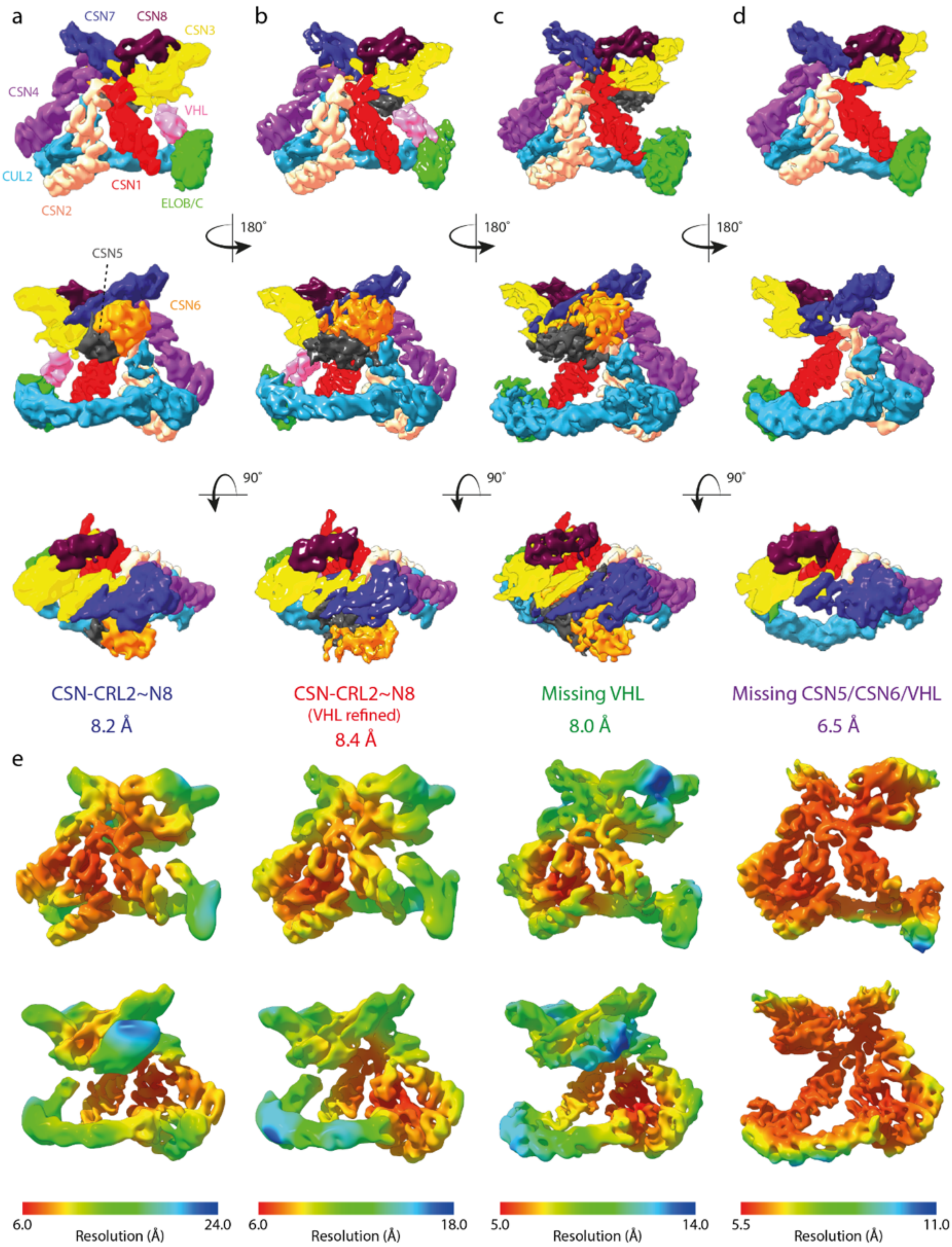


**Supplementary Figure 4. Cryo-EM structures of the CSN-CRL2~N8 segmented to highlight subunit composition.** Maps of the (**a**) CSN-CRL2~N8 holocomplex, (**b**) holocomplex (from VHL focused refinement), (**c**) holocomplex missing VHL (**d**) holocomplex

missing CSN5/CSN6 and VHL, are shown in various orientations. (**e**) Resolution maps of (**a-d**) from front and back views. Map resolutions generated using RELION[1] software.

a

b

c

d

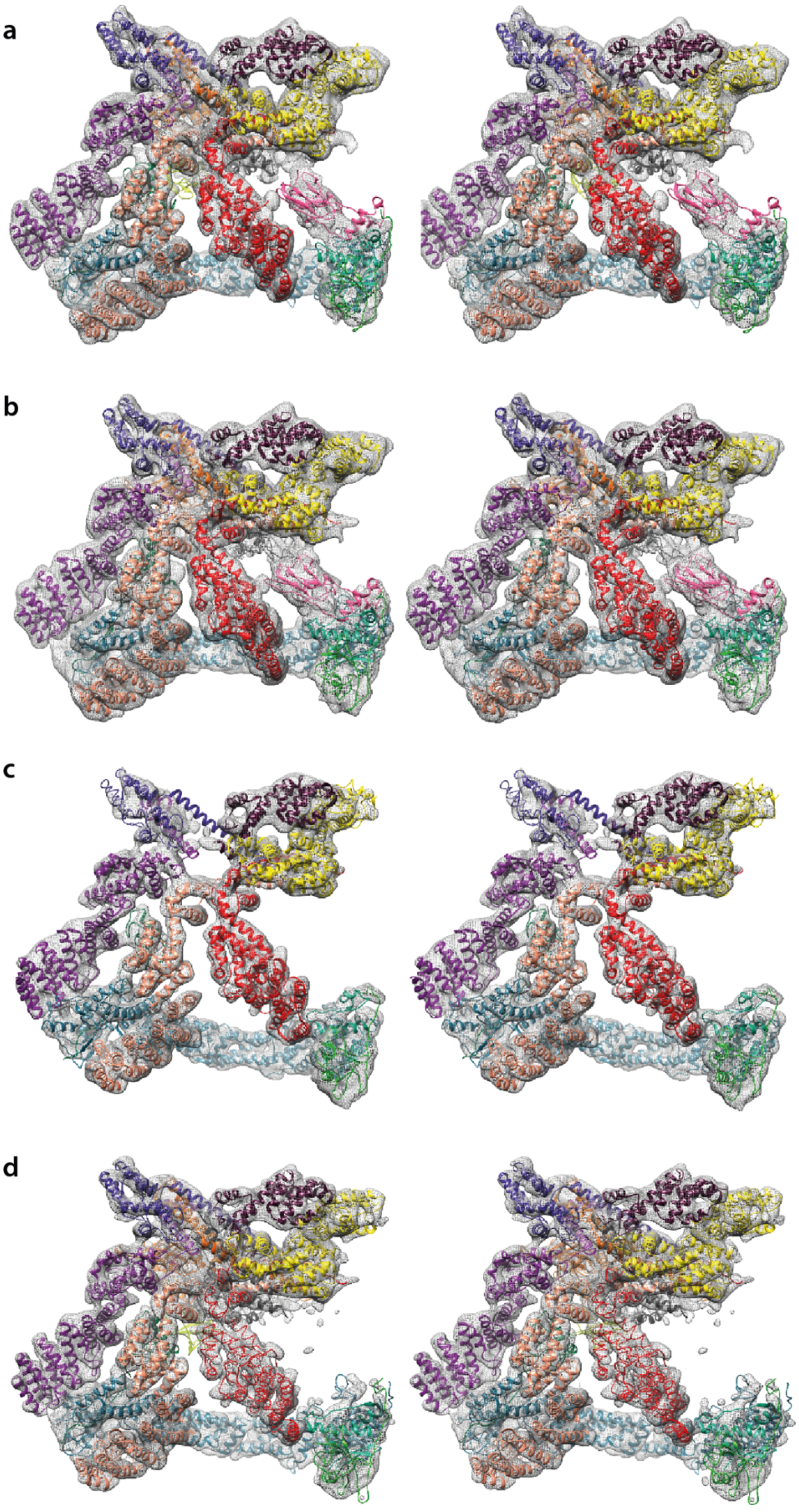

**Supplementary Figure 5**. Stereo images of CSN-CRL2~N8 complexes. Maps of the (**a**) CSN-CRL2~N8 holocomplex (threshold 0.022), (**b**) holocomplex (from VHL focused refinement;

threshold 0.014), (**c**) holocomplex missing VHL (threshold 0.014) and (**d**) holocomplex missing CSN5/CSN6 and VHL (threshold 0.014).

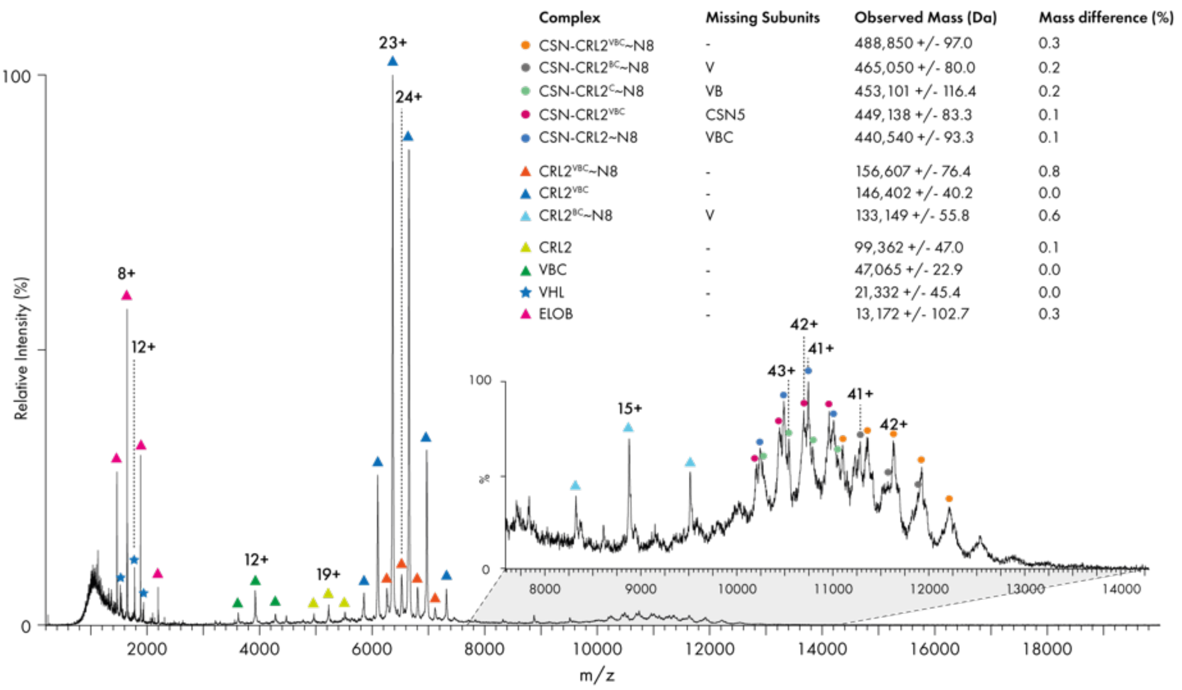


**Supplementary Figure 6. Native MS of the CSN-CRL2~N8 complex.** Spectra were collected from a 1:1 ratio of CSN and CRL2~N8, following a 1-hour incubation at room temperature and de-salting into 150 mM ammonium acetate (pH 7.5). The mass of each specie was assigned using Waters MassLynx (4.1). The identity, charge, observed mass and percentage mass difference (compared to the expected mass) is shown for each complex. CSN3 and CSN5 subunits included a 2x StrepII and 6HIS N-terminal tags, respectively. The mass of all CSN complexes identified in the spectra include the tag masses of CSN3 and CSN5. The presence of the VBC complex on CRL2 has been explicitly marked, including any missing subunits from subcomplexes. Details of data can be found in Supplementary Data 1.

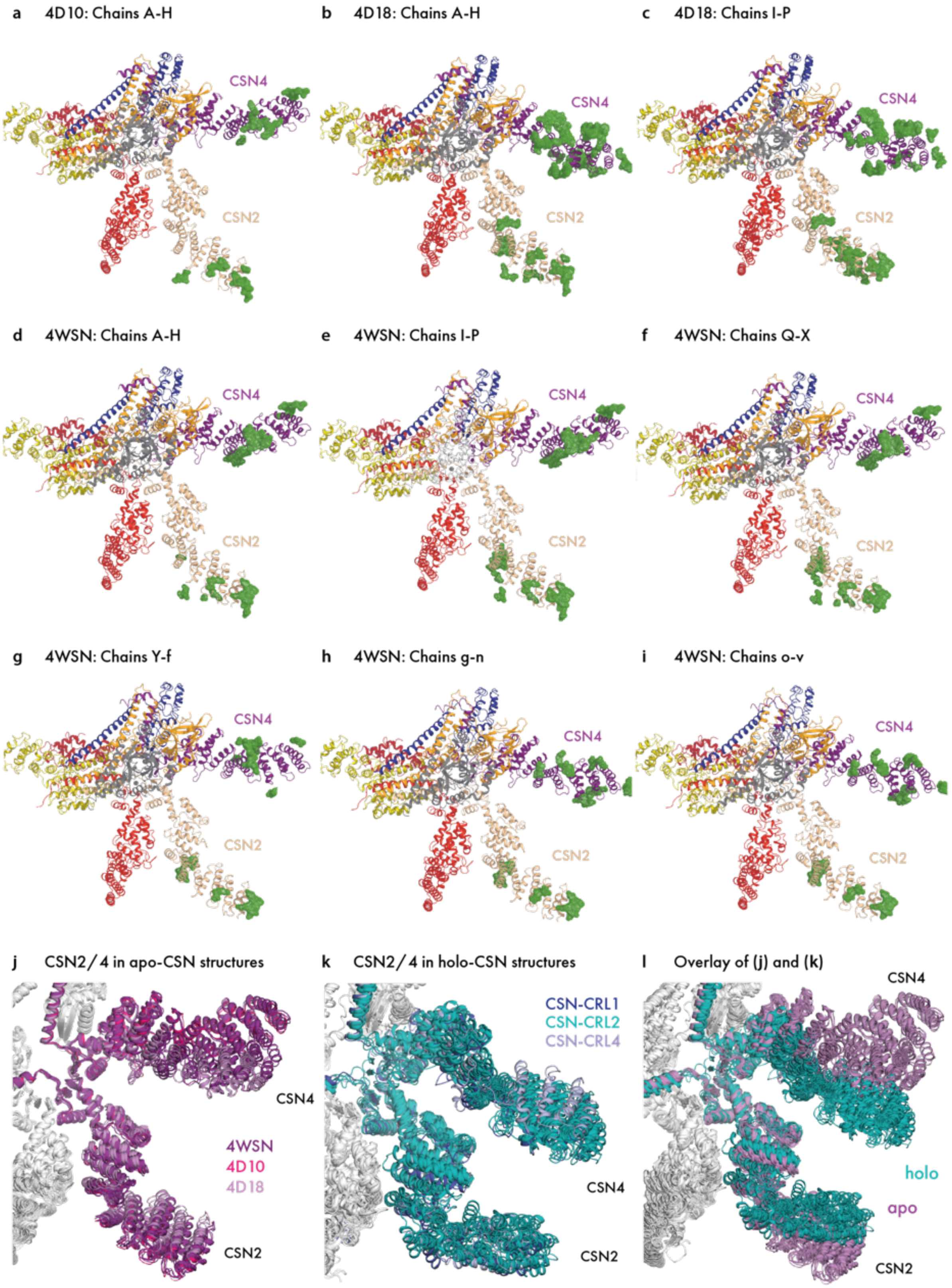


**Supplementary Figure 7. Conformations of CSN2 and CSN4 in apo and holo CSN. (a-i)** Conformations and crystal contacts of CSN2 and CSN4 for the nine independent molecules of apo-CSN in PDBs 4WSN, 4D10 and 4D18. Crystal contacts of CSN2 and CSN4 and

neighbouring asymmetric units within 5 Å are shown by the green mesh. **(j)** Overlay of apo-CSN molecules in (a-i) showing range of CSN2 and CSN4 conformations. **(k)** Overlay of holo-CSN molecules in cryo-EM structures of CSN-CRL1 (dark blue), CSN-CRL2 (teal) and CSN-CRL4 (light blue). **(l)** Overlay of (j) and (k). CSN2 and CSN4 in apo and holo structures of the CSN represent two distinct clusters of conformations.

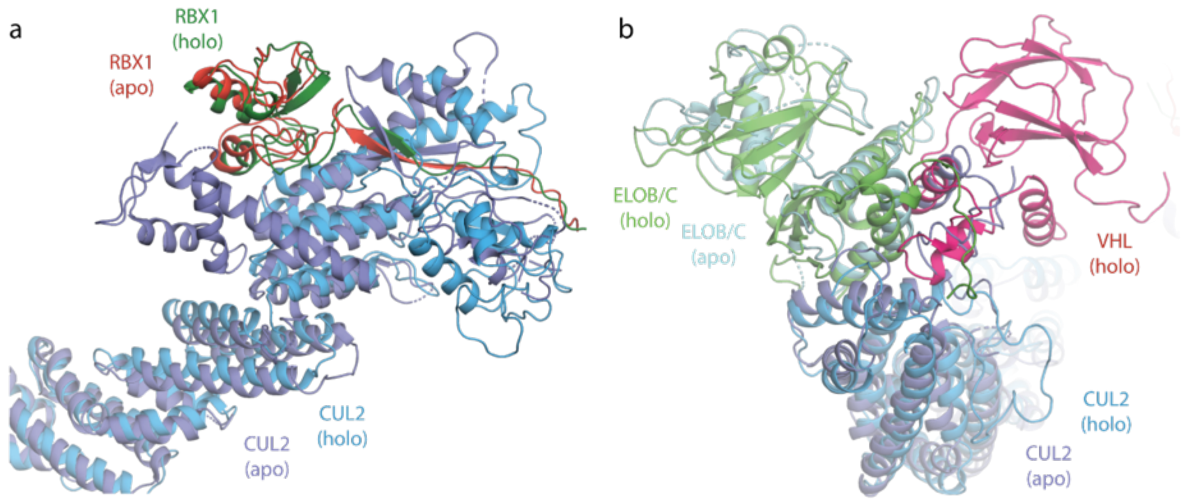


**Supplementary Figure 8.** Structural alignment of the CRL2 (**a**) C-terminal domain and (**b**) N-terminal domain in isolated (apo; 5N4W) and CSN-associated (holo) conformations. The structure of CSN has been hidden for clarity.

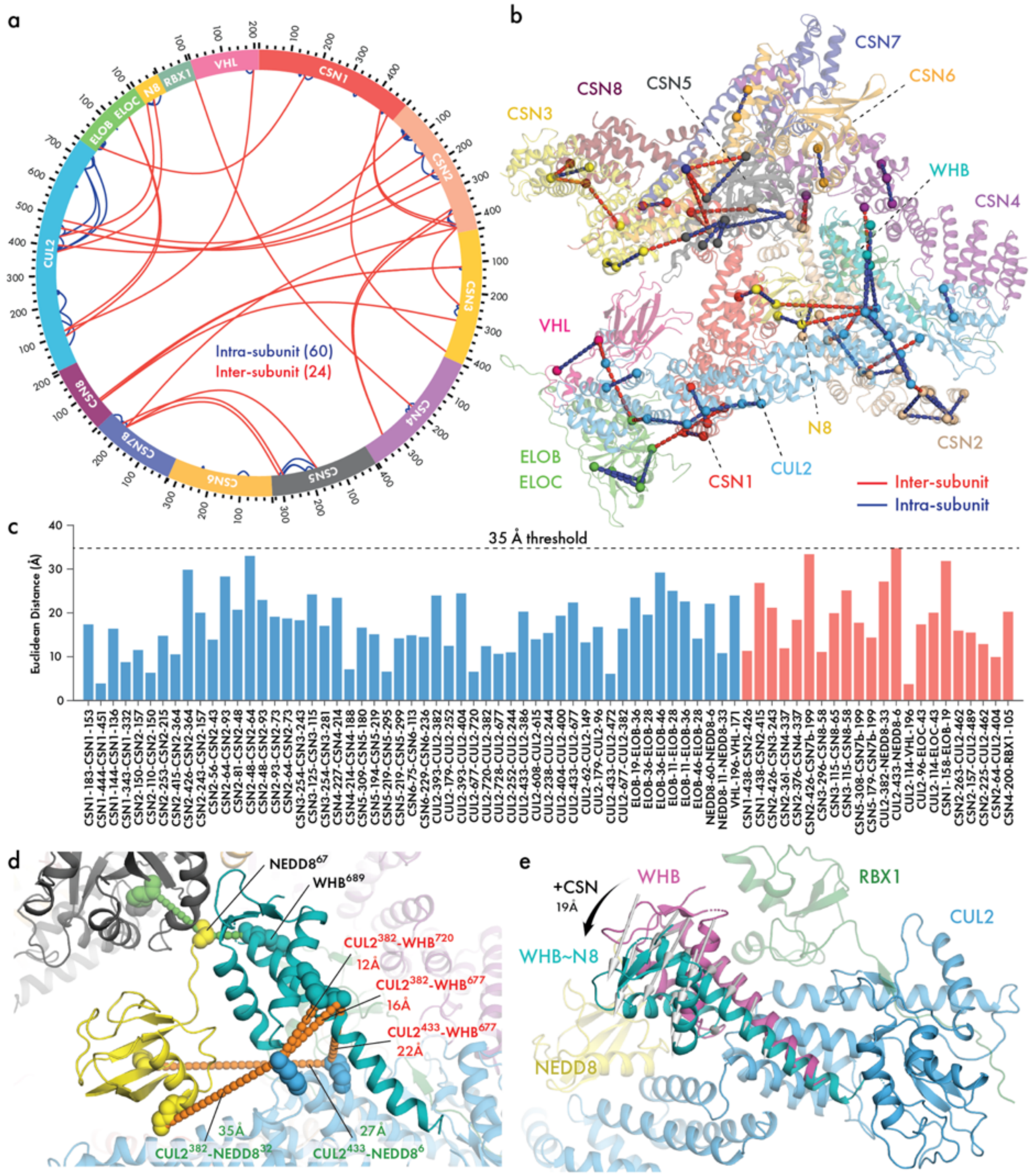


**Supplementary Figure 9. Cross-links of the CSN-CRL2~N8 complex.** (**a**) Circular plot of cross-links identified for the CSN-CRL2~N8 complex. (**b**) Inter- (red) and intra-subunit (blue) cross-links projected onto the cryo-EM structure of CSN-CRL2~N8. Nine cross-links were omitted due to missing residues in the C-terminal loops of CSN subunits (six) and self-residue cross-links (three) (**Supplementary Data 2**). (**c**) Euclidean distances for cross-links shown in (**b**). All measurements made between lysine Nζ-Nζ atoms using PyMOL. 35 Å cross-link distance threshold is shown by the dotted line. 35 Å takes into account two lysine sidechains

(15 Å), 10 Å cross-linker length for BS3 and an additional 10 Å to account for domain-level flexibility. (**d**) Zoom of the WHB~N8 region of the CSN-CRL2~N8 model generated from cryo-EM and cross-link. The WHB domain is shown in teal, CUL2 in light blue (transparent), NEDD8 in yellow and CSN5 in gray. (**e**) Comparison of WHB~N8 from our hybrid cryo-EM/cross-linking CSN-CRL2~N8 model (teal) with non-neddylated WHB from CRL2 crystal structure (5N4W; purple). Vector arrows depict the 19 Å movement of WHB following neddylation and binding to CSN. Source Data are provided as a Source Data file.

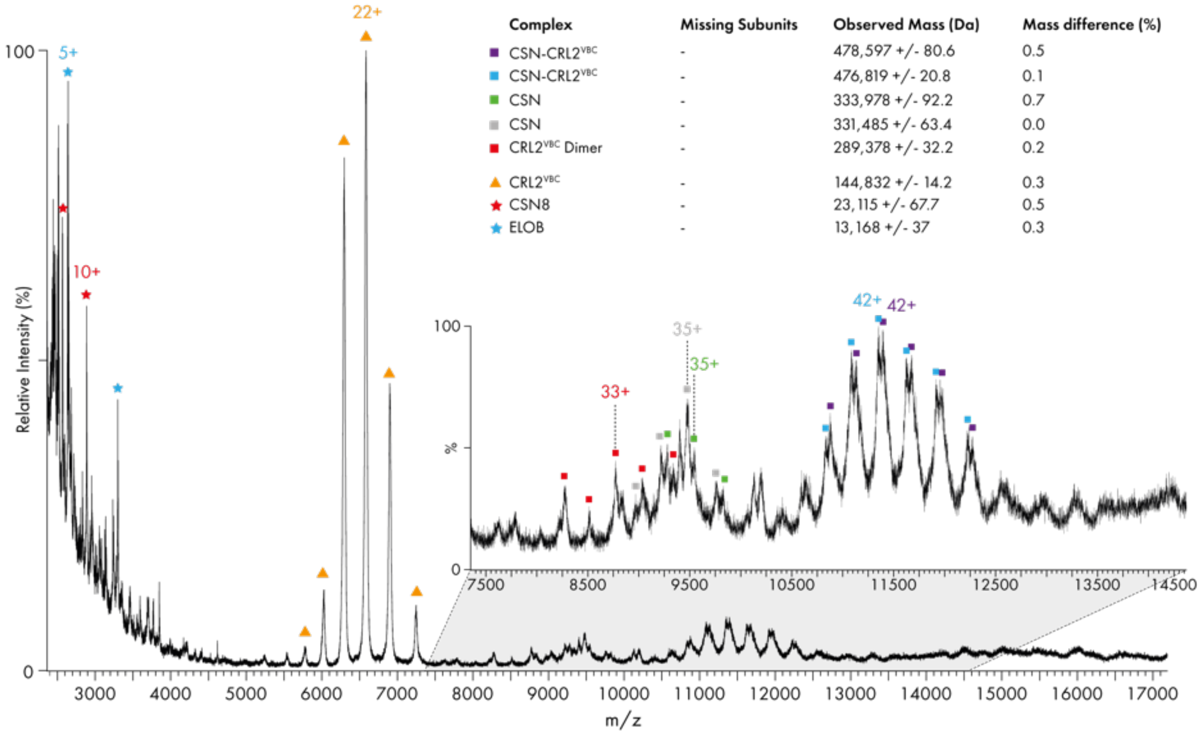


**Supplementary Figure 10. Native MS of the $CSN^{WT}$-CRL2.** Mass were assigned using Waters MassLynx software. Details of data can be found in Supplementary Data 1.

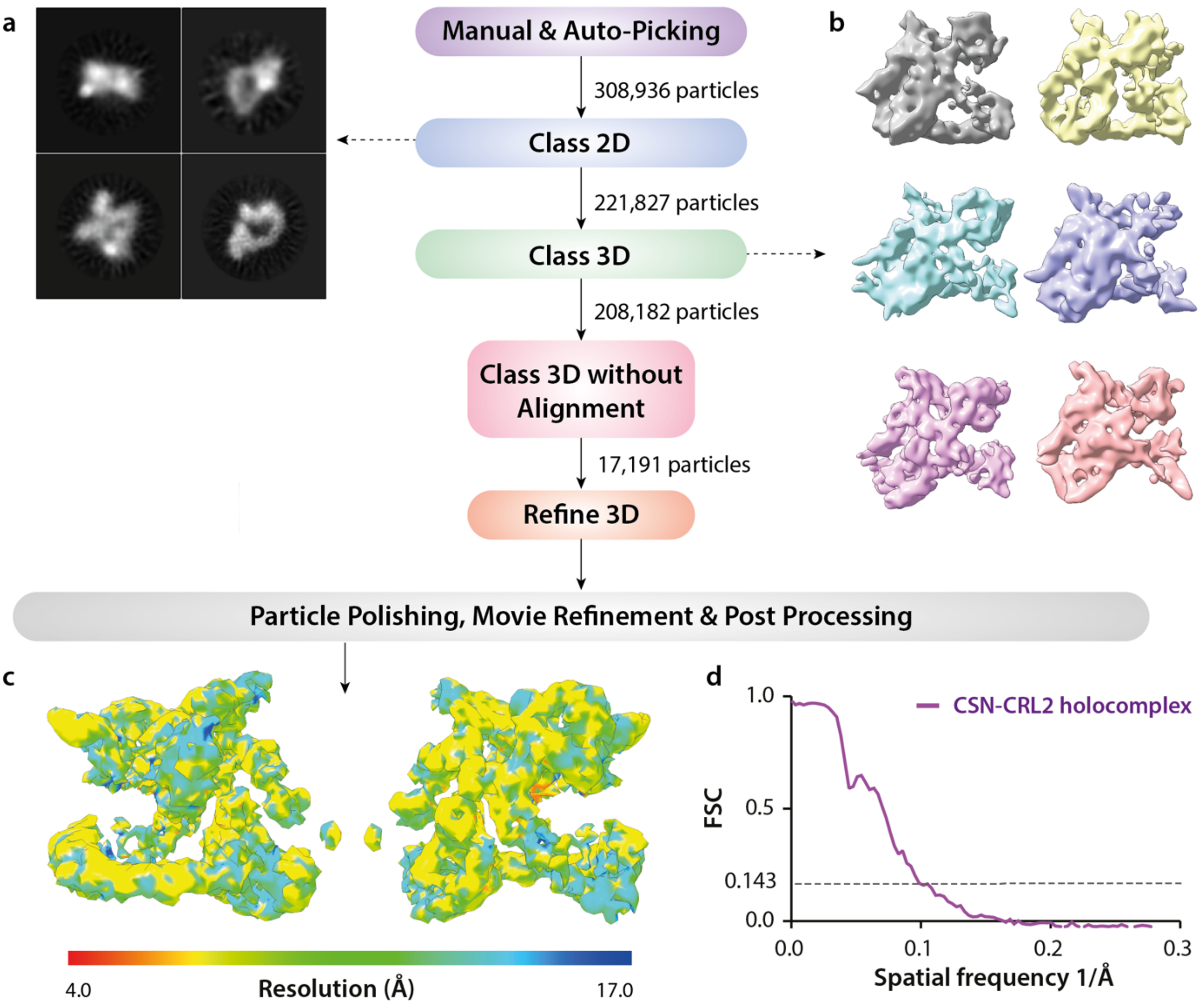


**Supplementary Figure 11. Cryo-EM map of the CSN-CRL2 complex.** A set of 6800 micrographs were subjected to manual and auto-picking in order to acquire particles for 2D reference-free classification (**a**). 2D classification was used for the positive selection of particles prior to 3D classification. (**b**) six classes generated, demonstrate subunit heterogeneity in the data set. (**c**) Resolution map of the CSN-CRL2 was generated using RELION[1]. (**d**) FSCs for the single CSN-CRL2 map. Source Data are provided as a Source Data file.

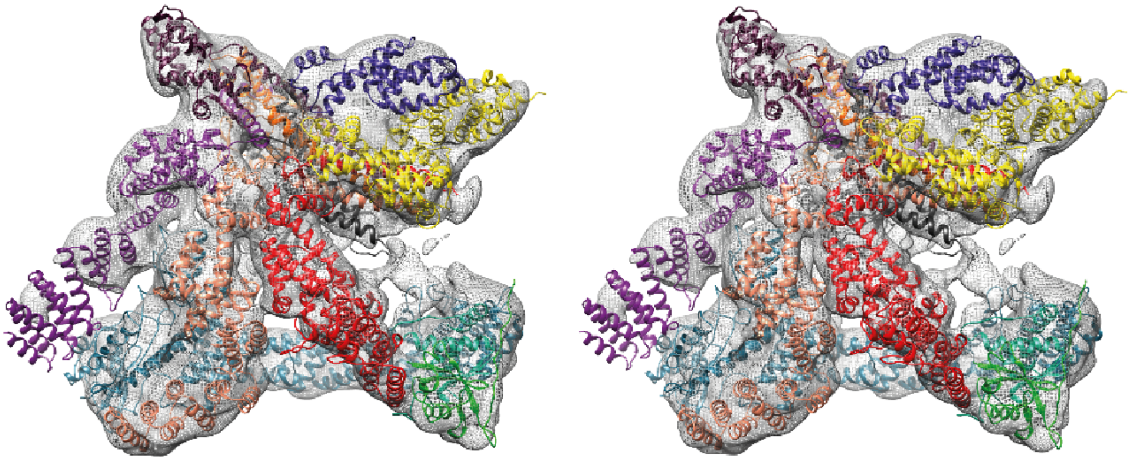

**Supplementary Figure 12**. Stereo images of the CSN-CRL2 complex (threshold 0.014).

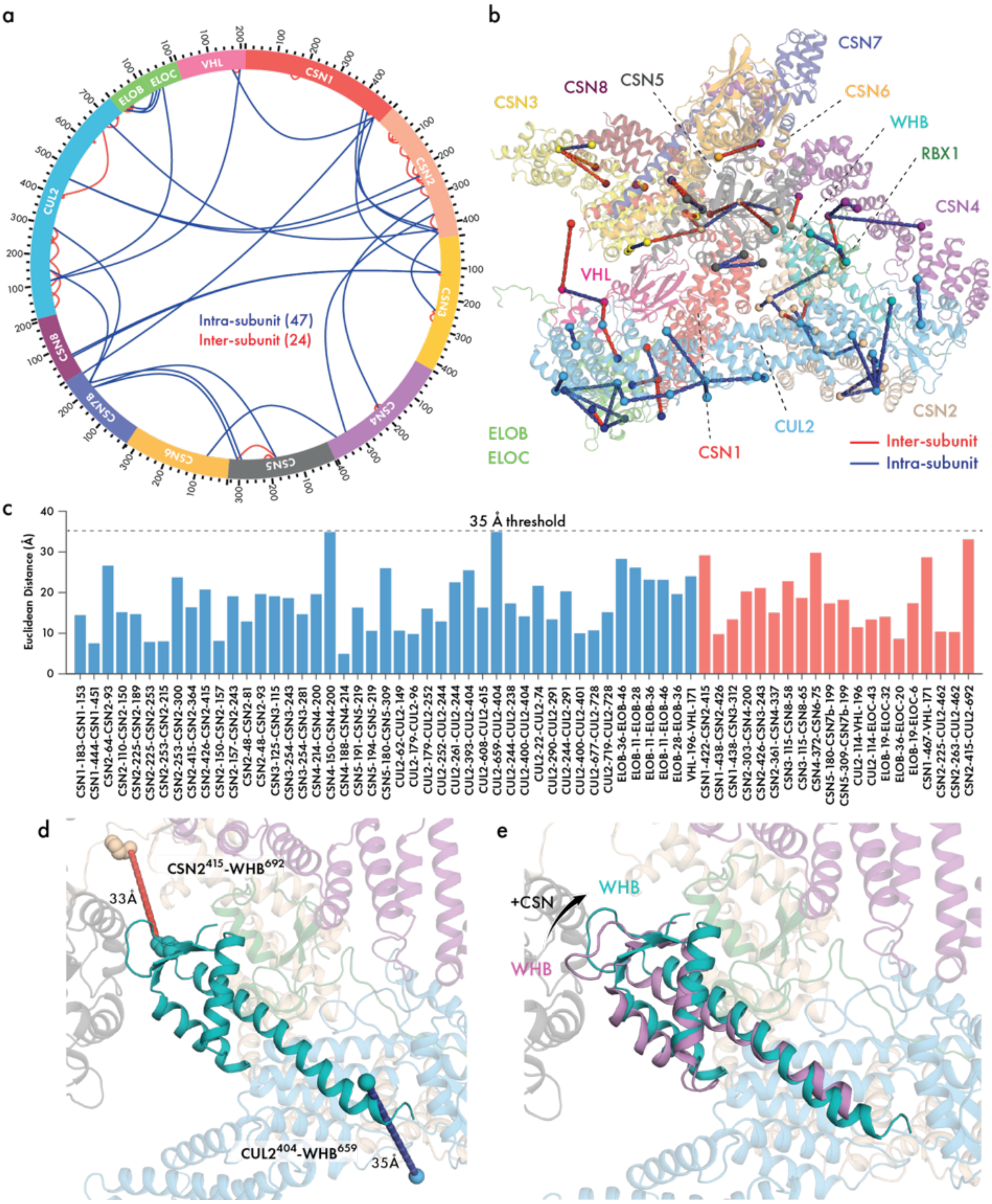


**Supplementary Figure 13. Cross-links of the deneddylated CSN-CRL2 complex.** (**a**) Circular plot of cross-links identified for the CSN-CRL2 complex. (**b**) Inter- (red) and intra-subunit (blue) cross-links projected onto the cryo-EM structure of CSN-CRL2. Six cross-links were omitted due to missing residues in the C-terminal loops of CSN subunits (five) and self-residue cross-links (one) (**Supplementary Data 3**). (**c**) Euclidean distances for cross-links

shown in (**b**). All measurements made between lysine Nζ-Nζ atoms using PyMOL. 35 Å cross-link distance threshold is shown by the dotted line. 35 Å takes into account two lysine sidechains (15 Å), 10 Å cross-linker length for BS3 and an additional 10 Å to account for domain-level flexibility. (**e**) Comparison of deneddylated WHB from our hybrid cryo-EM/cross-linking CSN-CRL2 model (teal) with WHB from CRL2 crystal structure (5N4W; purple). Source Data are provided as a Source Data file.

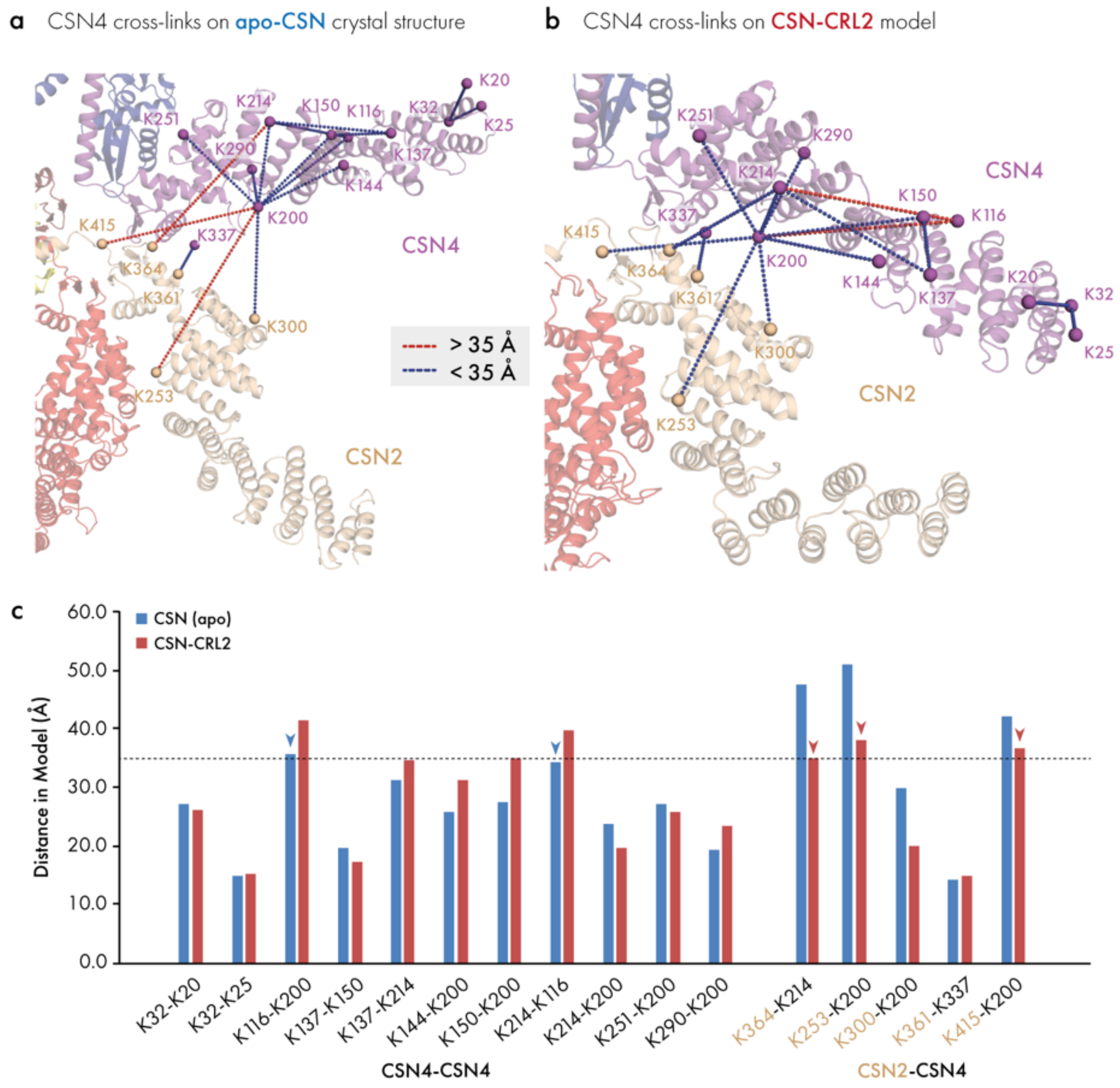


**Supplementary Figure 14. Cross-links of CSN4 from apo-CSN$^{WT}$.** Location and distances of CSN4 cross-links in apo-CSN$^{WT}$, projected onto the structures of (a) apo-CSN crystal structure (PDB 4D10) and (b) cryo-EM map fitted model of CSN-CRL2. Cross-links with distances satisfied under the cut-off of 35 Å are shown by dashed red lines, while those that are satisfied are in blue. (c) Bar plot showing distances of cross-links from (a-b). Dashed line represents a 35 Å distance threshold. Arrows mark cross-links which are satisfied in one conformation of the CSN but not the other. For K116-K200, K364-K214, K253-K200 and K415-K200 cross-links we included the shortest distance model in the satisfied category due to the distances being close to 35 Å, while the alternative conformation is much greater than 35 Å. All measurements were from lysine NZ atoms. The three CSN2-CSN4 cross links which

differentiate between open and closed models (red arrows) are each located relatively close to the hinge domain of CSN4, nevertheless the distance between lysine residues changes quite substantially. Although potential cross links between residues closer to the N-termini of CSN2 and CSN4 would undergo a significantly greater change in separation, they were not observed and would not be expected to occur with the cross-linking reagent used here since the separation is substantially greater than 35 Å for both open and closed conformations. Source Data are provided as a Source Data file.

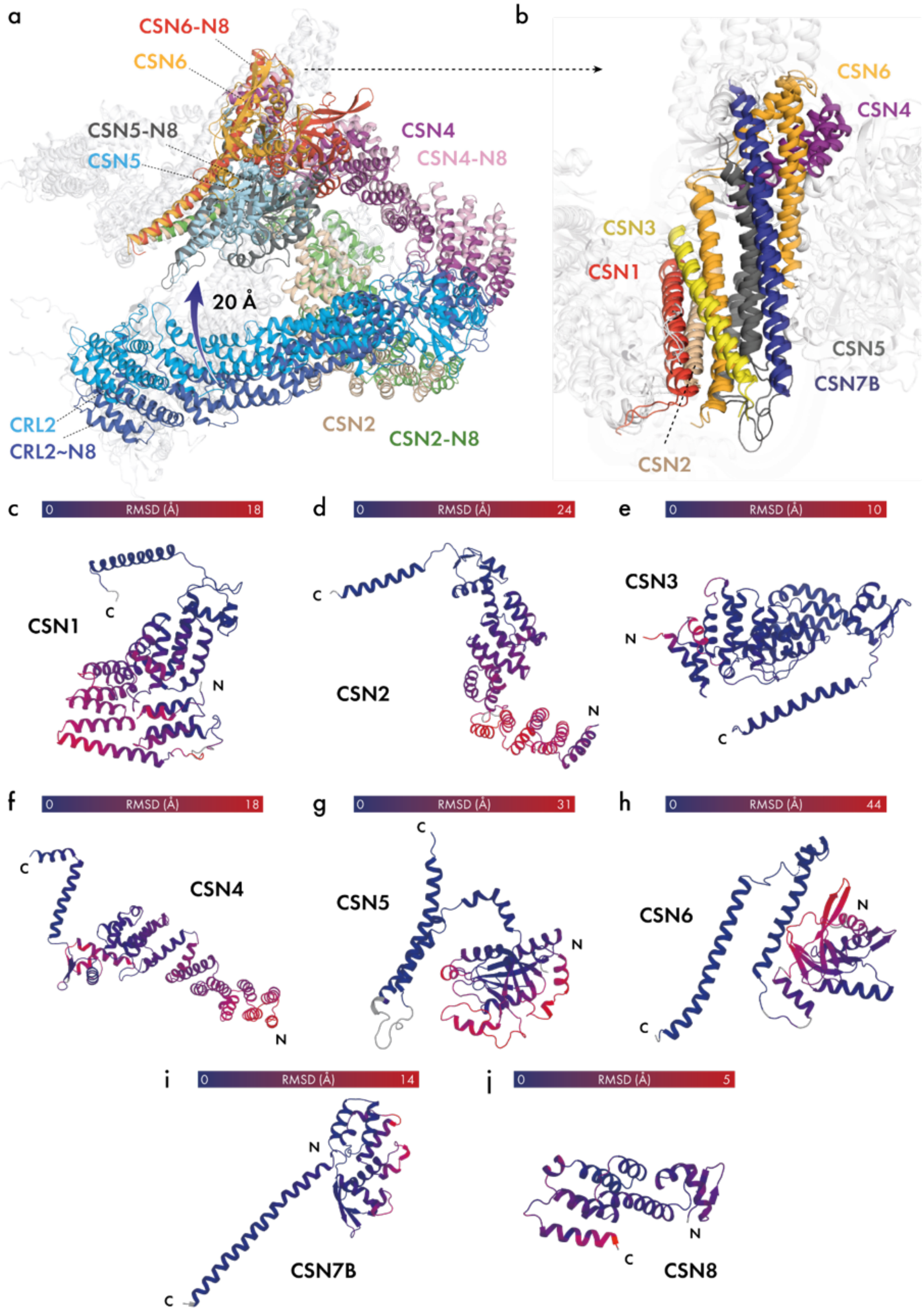


**Supplementary Figure 15. Per-subunit comparisons of the CSN in neddylated and non-neddylated CSN-CRL2 complexes.** (**a**) Superposition of the CSN-CRL2~N8 and CSN-

CRL2. CSN1, CSN3, CSN7B, CSN8, RBX1, ELOB/C and VHL are shown in white to highlight the changes in CSN5/CSN6, CSN2/CSN4 and Cullin-2. Cullin-2 rotates upwards by ~20 Å in the absence of NEDD8. (**b**) Alignment of the C-terminal helical bundle of CSN-CRL2 and CSN-CRL2~N8 complexes. Per-subunit comparisons of (**c**) CSN1, (**d**) CSN2, (**e**) CSN3, (**f**), CSN4, (**g**) CSN5, (**h**) CSN6, (**i**) CSN7B, (**j**) CSN8 between CSN-CRL2~N8 and CSN-CRL2 structures. The per-residue RMSD of each CSN1-8 subunit between CSN-CRL2~N8 and CSN-CRL2 complexes are indicated by the blue-red gradient. Each colour bar and colour gradient has been normalised to the maximum RMSD calculated for that subunit. The structure shown is the non-neddylated CSN-CRL2. The maximum per-subunit RMSDs measured were: CSN1 17.6 Å, CSN2 23.6 Å, CSN3 9.6 Å, CSN4 17.6 Å, CSN5 30.6 Å, CSN6 44.3 Å, CSN7B 13.5 Å, CSN8 5 Å.

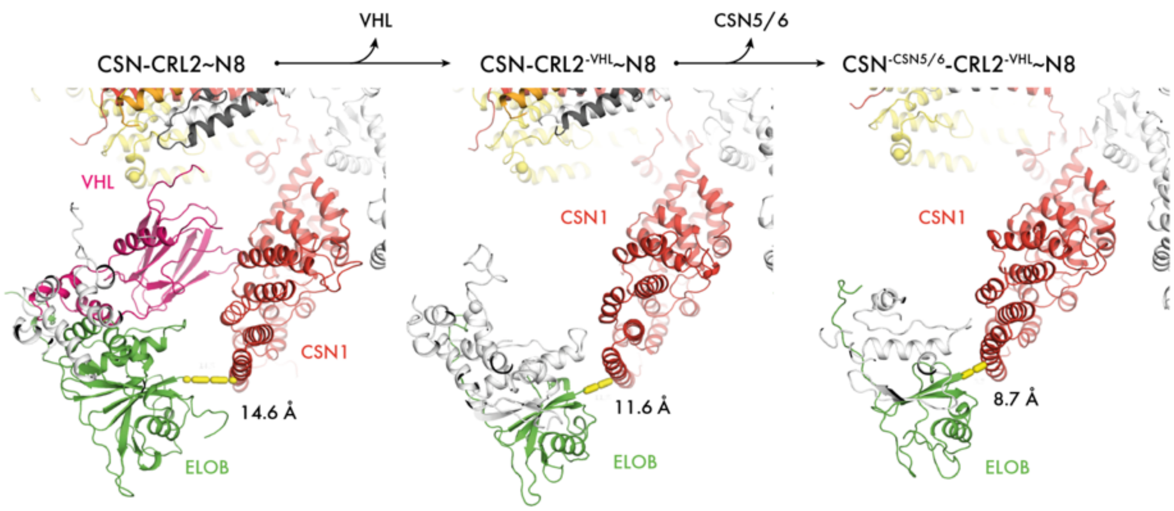


**Supplementary Figure 16. CSN1-ELOB interface in CSN-CRL2~N8 complexes.** The distance between CSN1 and ELOB of CSN-CRL2~N8, after loss of VHL and after loss of CSN5/CSN6 is shown by the dashed yellow line. All other CSN-CRL2 subunits have been coloured in white for clarity.

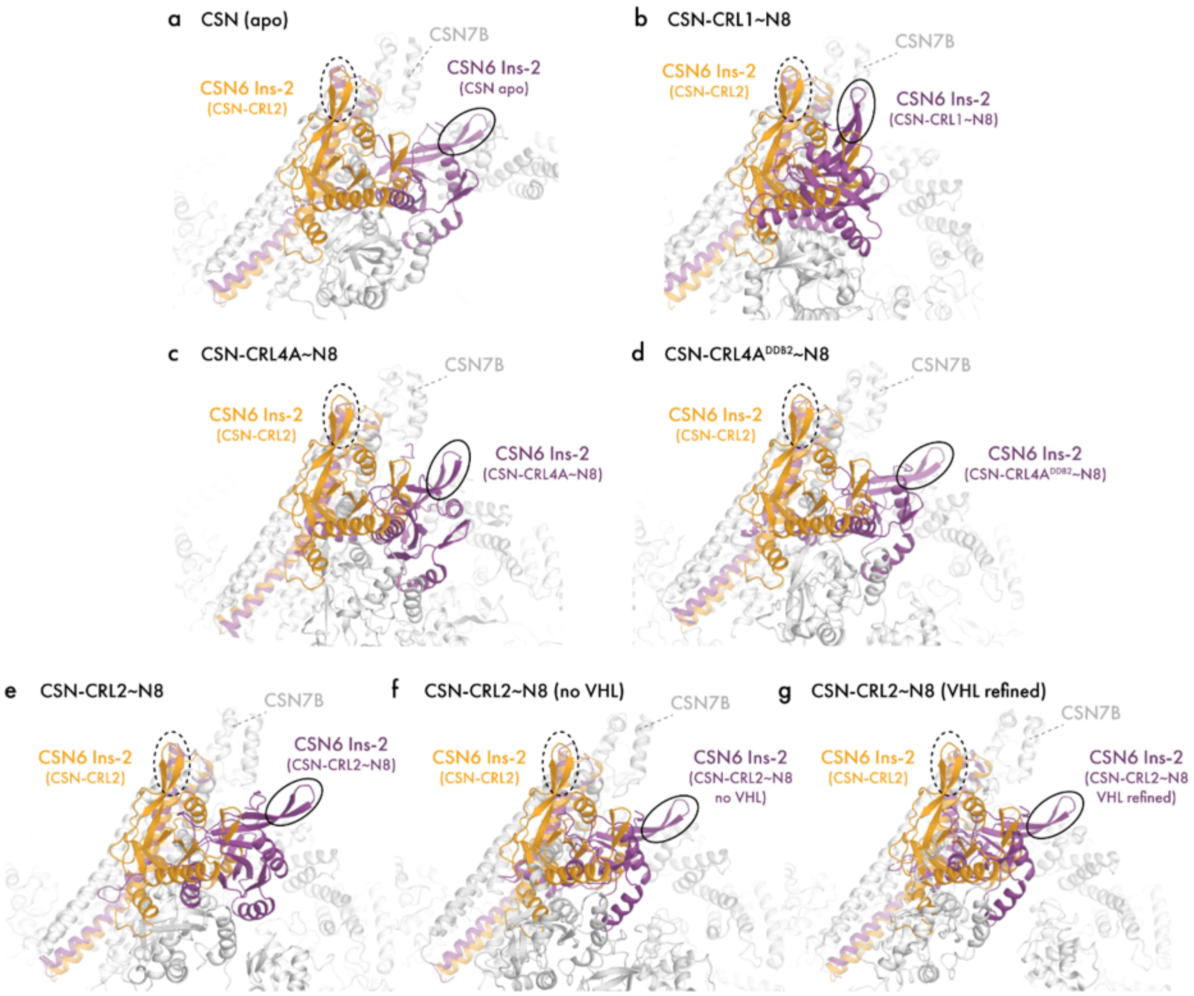


**Supplementary Figure 17. Comparison of CSN6 conformations in published CSN and CSN-CRL complexes.** Alignment of CSN-CRL2 (orange) with (a) crystal structure of apo CSN (PDB 4D10), (b) CSN-CRL1~N8 (EMD-3401), (c-d) fitted coordinates of CSN-CRL4A~N8 (EMD-3315) and CSN-CRL4A$^{DDB2}$~N8 (EMD-3316), (e-g) fitted coordinates of neddylated CSN-CRL2~N8 intact complex, missing VHL and VHL-refined (all purple). The CSN6 Ins-2 loop for each CSN6 has been highlighted for clarity (CSN6 of CSN-CRL2 with dashed ellipses, CSN6 of all other complexes in solid ellipses). Numerical quantification of similarity between CSN6 conformations is presented in Supplementary Figure 13.

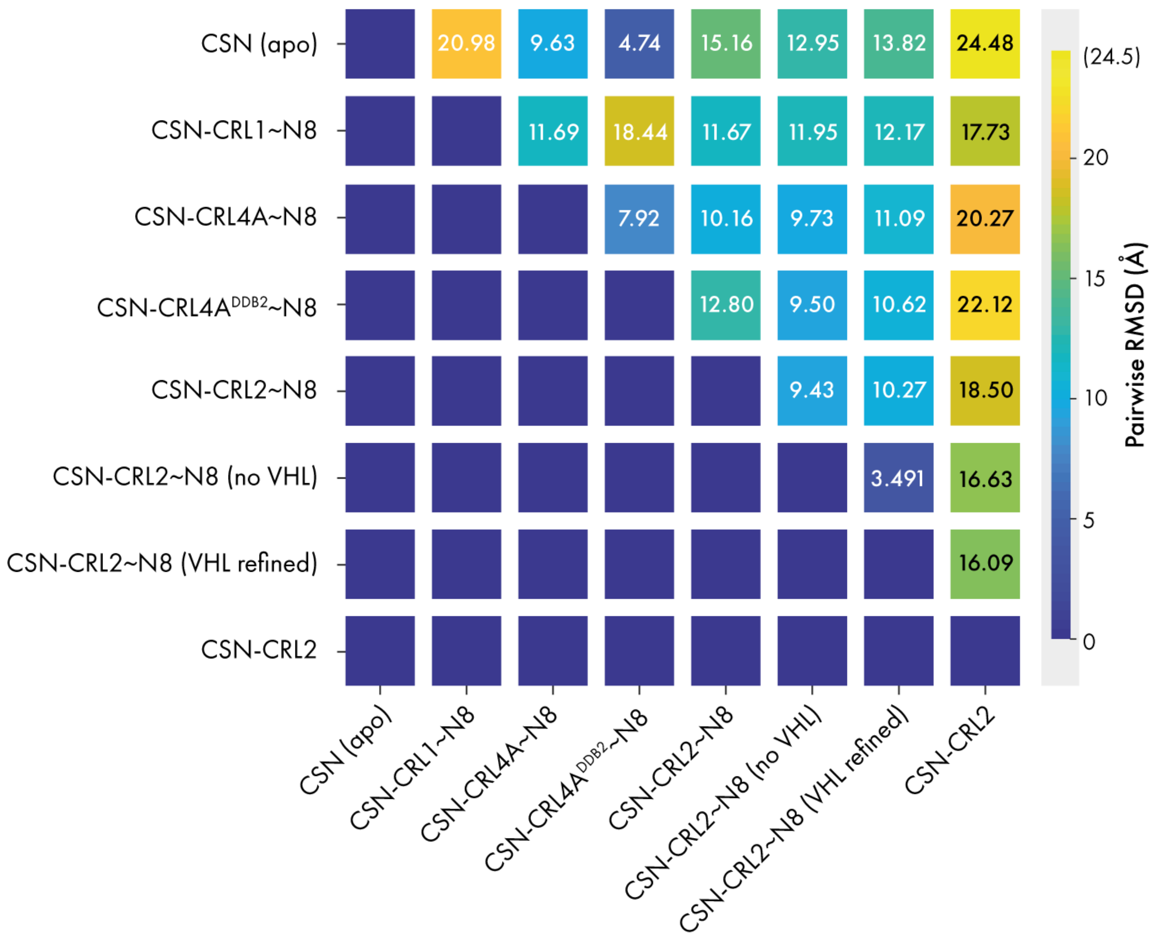


**Supplementary Figure 18. Pairwise RMSD matrix of CSN6 in CSN and CSN-CRL complexes.** Matrix indicates the RMSD between comparisons of CSN6 combinations in the apo CSN, CSN-CRL1~N8 (EMD-3401), CSN-CRL4A~N8 (EMD-3315), CSN-CRL4A$^{DDB2}$~N8 (EMD-3316), CSN-CRL2~N8, CSN-CRL2~N8 (-VHL), CSN-CRL2~N8 (VHL refined) and non-neddylated CSN-CRL2 complexes. The CSN1, CSN2, CSN3, CSN4, CSN7 and CSN8 subunits were aligned in each comparison and the RMSD was calculated between the non-fitted coordinates of CSN6 in each alignment using PyMOL. Aligned models are shown in Supplementary Figure 12.

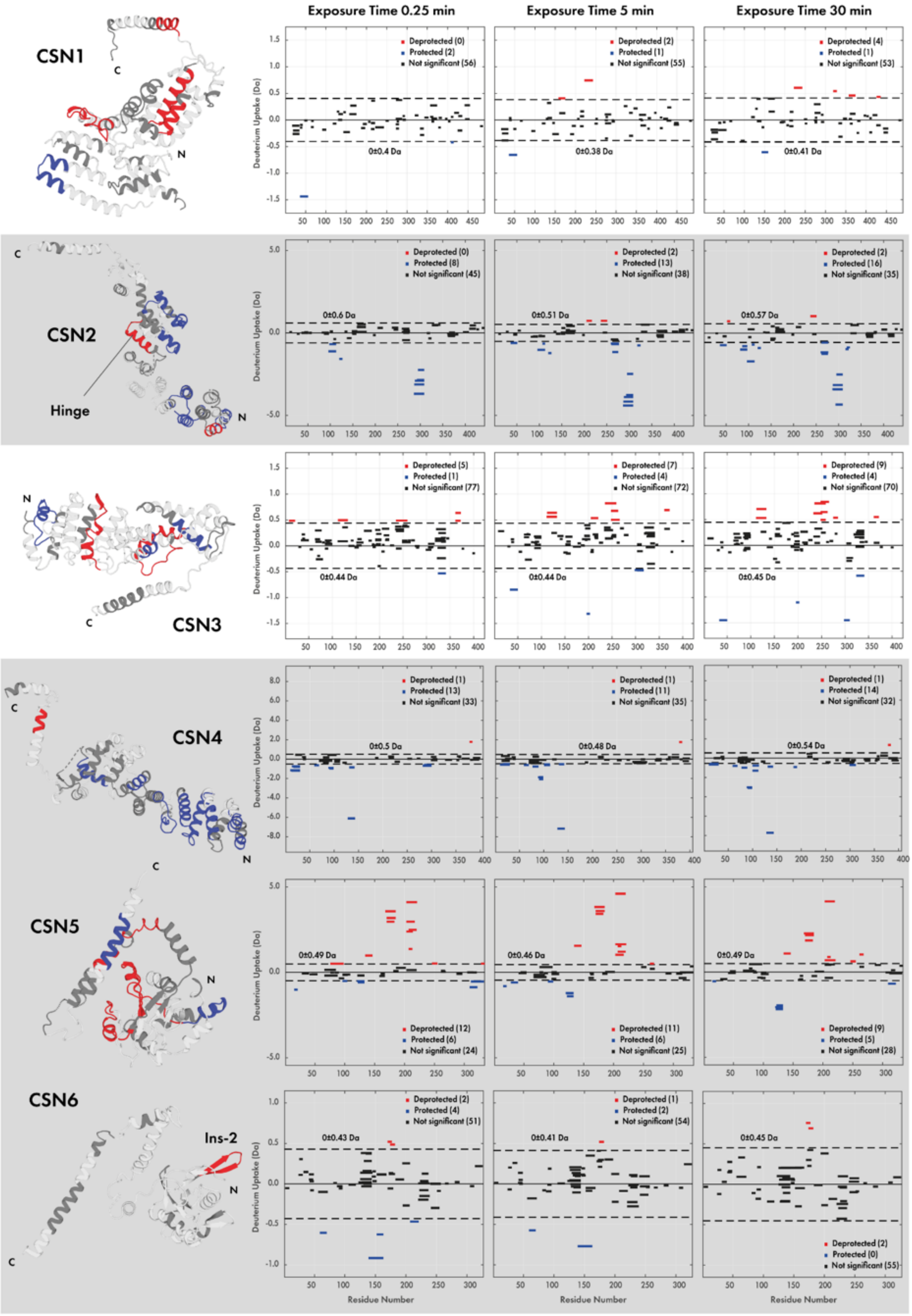
Exposure Time 0.25 min
Exposure Time 5 min
Exposure Time 30 min
CSN1
CSN2
Hinge
CSN3
CSN4
CSN5
CSN6
Ins-2
Deuterium Uptake (Da)
Residue Number
Deprotected (0)
Protected (2)
Not significant (56)
0±0.4 Da
Deprotected (2)
Protected (1)
Not significant (55)
0±0.38 Da
Deprotected (4)
Protected (1)
Not significant (53)
0±0.41 Da
Deprotected (0)
Protected (8)
Not significant (45)
0±0.6 Da
Deprotected (2)
Protected (13)
Not significant (38)
0±0.51 Da
Deprotected (2)
Protected (16)
Not significant (35)
0±0.57 Da
Deprotected (5)
Protected (1)
Not significant (77)
0±0.44 Da
Deprotected (7)
Protected (4)
Not significant (72)
0±0.44 Da
Deprotected (9)
Protected (4)
Not significant (70)
0±0.45 Da
Deprotected (1)
Protected (13)
Not significant (33)
0±0.5 Da
Deprotected (1)
Protected (11)
Not significant (35)
0±0.48 Da
Deprotected (1)
Protected (14)
Not significant (32)
0±0.54 Da
Deprotected (12)
Protected (6)
Not significant (24)
0±0.49 Da
Deprotected (11)
Protected (6)
Not significant (25)
0±0.46 Da
Deprotected (9)
Protected (5)
Not significant (28)
0±0.49 Da
Deprotected (2)
Protected (4)
Not significant (51)
0±0.43 Da
Deprotected (1)
Protected (2)
Not significant (54)
0±0.41 Da
Deprotected (2)
Protected (0)
Not significant (55)
0±0.45 Da

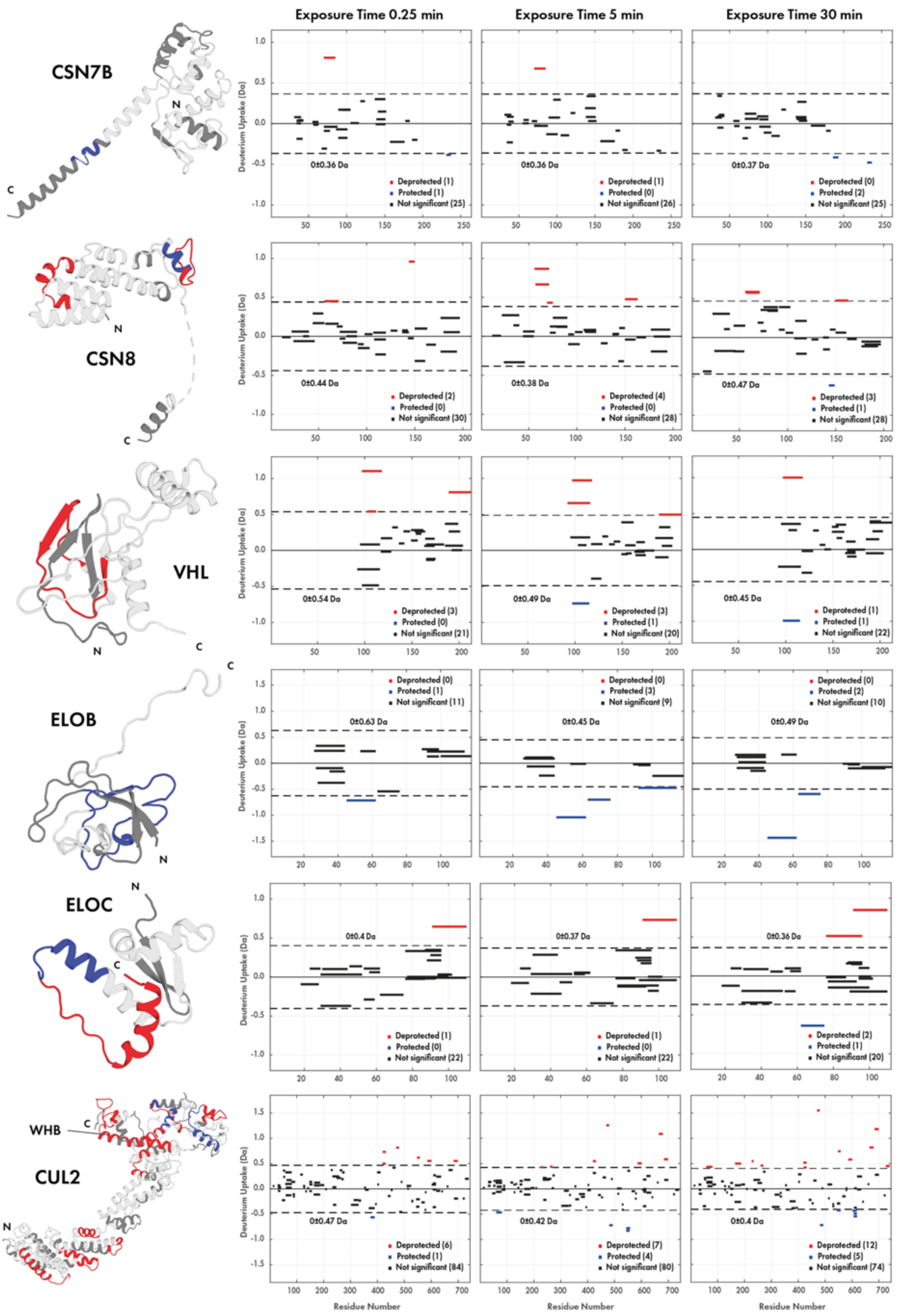
Exposure Time 0.25 min
Exposure Time 5 min
Exposure Time 30 min
CSN7B
CSN8
VHL
ELOB
ELOC
CUL2
WHB
N
C
Deuterium Uptake (Da)
Residue Number
0±0.36 Da
Deprotected (1)
Protected (1)
Not significant (25)
0±0.36 Da
Deprotected (1)
Protected (0)
Not significant (26)
0±0.37 Da
Deprotected (0)
Protected (2)
Not significant (25)
0±0.44 Da
Deprotected (2)
Protected (0)
Not significant (30)
0±0.38 Da
Deprotected (4)
Protected (0)
Not significant (28)
0±0.47 Da
Deprotected (3)
Protected (1)
Not significant (28)
0±0.54 Da
Deprotected (3)
Protected (0)
Not significant (21)
0±0.49 Da
Deprotected (3)
Protected (1)
Not significant (20)
0±0.45 Da
Deprotected (1)
Protected (1)
Not significant (22)
0±0.63 Da
Deprotected (0)
Protected (1)
Not significant (11)
0±0.45 Da
Deprotected (0)
Protected (3)
Not significant (9)
0±0.49 Da
Deprotected (0)
Protected (2)
Not significant (10)
0±0.4 Da
Deprotected (1)
Protected (0)
Not significant (22)
0±0.37 Da
Deprotected (1)
Protected (0)
Not significant (22)
0±0.36 Da
Deprotected (2)
Protected (1)
Not significant (20)
0±0.47 Da
Deprotected (6)
Protected (1)
Not significant (84)
0±0.42 Da
Deprotected (7)
Protected (4)
Not significant (80)
0±0.4 Da
Deprotected (12)
Protected (5)
Not significant (74)

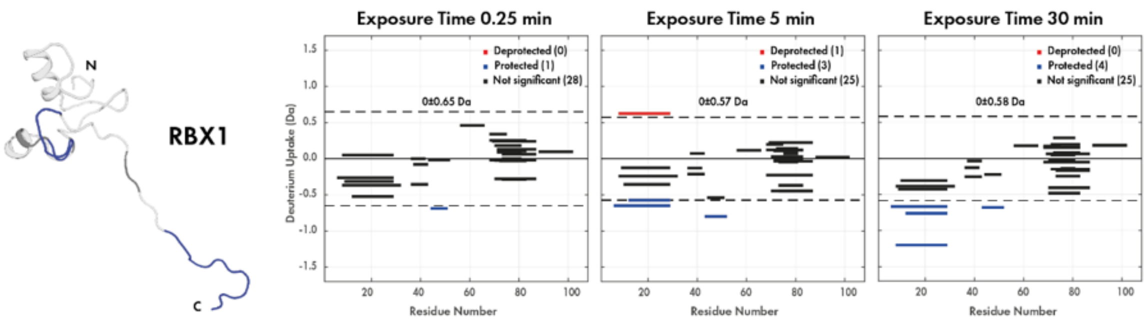


**Supplementary Figure 19. HDX-MS of CSN-CRL2~N8 per protein per timepoint.** Differential comparison of Δ(CSN-CRL2~N8 – CSN). Peptides experiencing stabilisation upon CRL2~N8 binding to CSN, compared to the peptide in apo CSN, are shown as blue, destabilised peptides are shown in red. CSN2, CSN4, CSN5 and CSN6 have been highlighted by gray boxes. The CSN2 hinge and Cullin-2 WHB domain have been highlighted in grey for clarity. Peptides were filtered applying a 98% confidence limit (critical value of 6.965; dotted lines). Structures show data for the 30 min timepoint. Details of data can be found in Supplementary Data 1.

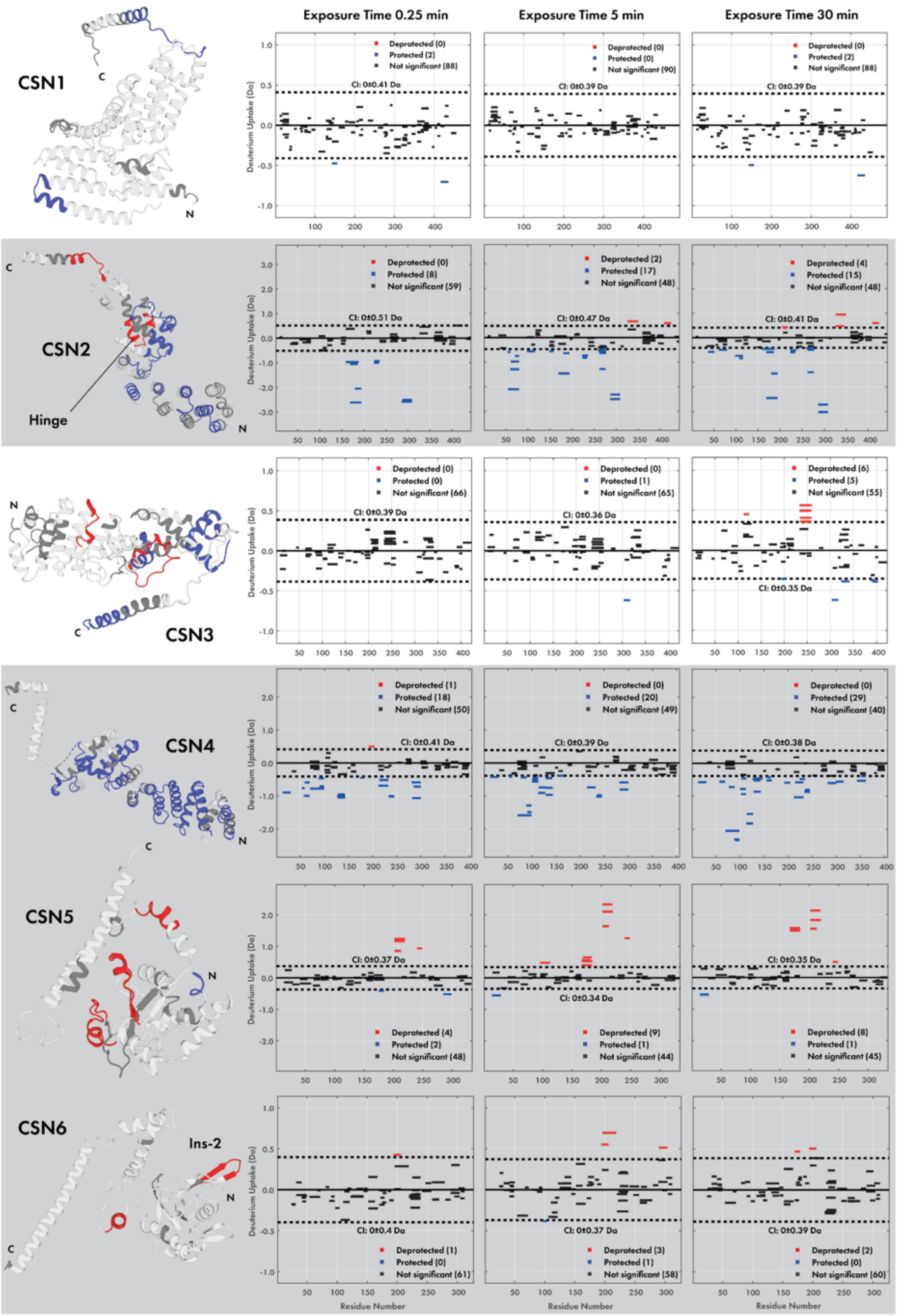
Exposure Time 0.25 min
Exposure Time 5 min
Exposure Time 30 min
CSN1
CSN2
Hinge
CSN3
CSN4
CSN5
CSN6
Ins-2
Deuterium Uptake (Da)
Residue Number
Deprotected
Protected
Not significant

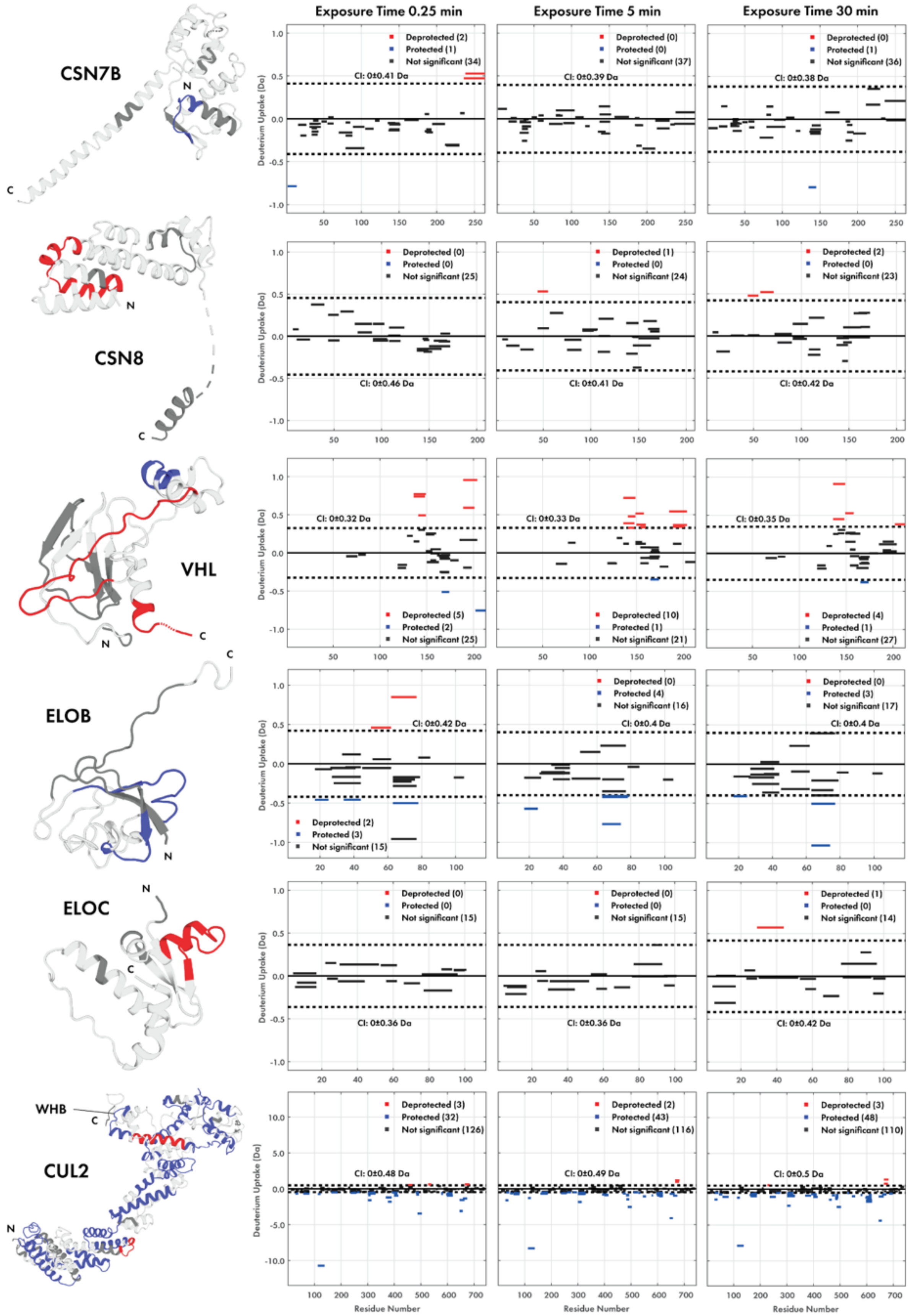

Exposure Time 0.25 min
Exposure Time 5 min
Exposure Time 30 min
CSN7B
CSN8
VHL
ELOB
ELOC
CUL2
WHB
N
C
Deprotected (2)
Protected (1)
Not significant (34)
CI: 0±0.41 Da
Deprotected (0)
Protected (0)
Not significant (37)
CI: 0±0.39 Da
Deprotected (0)
Protected (1)
Not significant (36)
CI: 0±0.38 Da
Deprotected (0)
Protected (0)
Not significant (25)
CI: 0±0.46 Da
Deprotected (1)
Protected (0)
Not significant (24)
CI: 0±0.41 Da
Deprotected (2)
Protected (0)
Not significant (23)
CI: 0±0.42 Da
CI: 0±0.32 Da
Deprotected (5)
Protected (2)
Not significant (25)
CI: 0±0.33 Da
Deprotected (10)
Protected (1)
Not significant (21)
CI: 0±0.35 Da
Deprotected (4)
Protected (1)
Not significant (27)
CI: 0±0.42 Da
Deprotected (2)
Protected (3)
Not significant (15)
Deprotected (0)
Protected (4)
Not significant (16)
CI: 0±0.4 Da
Deprotected (0)
Protected (3)
Not significant (17)
CI: 0±0.4 Da
Deprotected (0)
Protected (0)
Not significant (15)
CI: 0±0.36 Da
Deprotected (0)
Protected (0)
Not significant (15)
CI: 0±0.36 Da
Deprotected (1)
Protected (0)
Not significant (14)
CI: 0±0.42 Da
Deprotected (3)
Protected (32)
Not significant (126)
CI: 0±0.48 Da
Deprotected (2)
Protected (43)
Not significant (116)
CI: 0±0.49 Da
Deprotected (3)
Protected (48)
Not significant (110)
CI: 0±0.5 Da
Deuterium Uptake (Da)
Residue Number

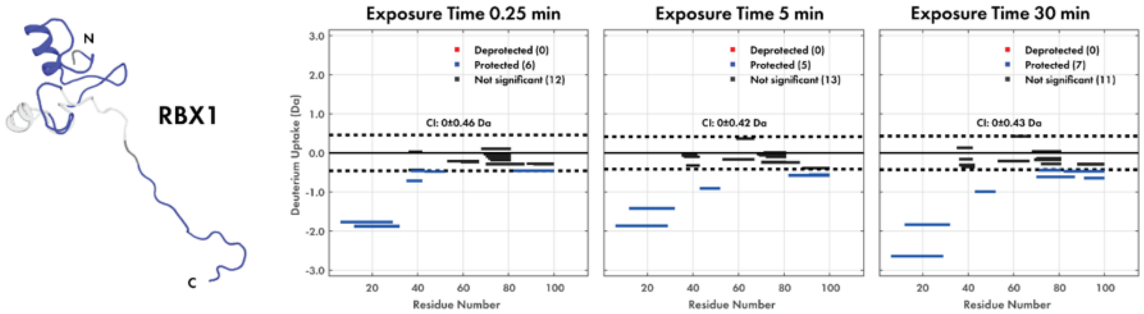


**Supplementary Figure 20. HDX-MS of CSN$^{WT}$-CRL2 per protein per timepoint.** Differential comparison of Δ(CSN$^{WT}$-CRL2 – CSN$^{WT}$). Peptides experiencing stabilisation upon non-neddylated CRL2 binding to CSN$^{WT}$, compared to the peptide in apo CSN$^{WT}$, are shown as blue, destabilised peptides are shown in red. CSN2, CSN4, CSN5 and CSN6 have been highlighted by gray boxes. The CSN2 hinge and Cullin-2 WHB domain have been highlighted for clarity. Peptides were filtered applying a 98% confidence limit (critical value of 6.965; dotted lines). Structures show data for the 30 min timepoint. Details of data can be found in Supplementary Data 1.

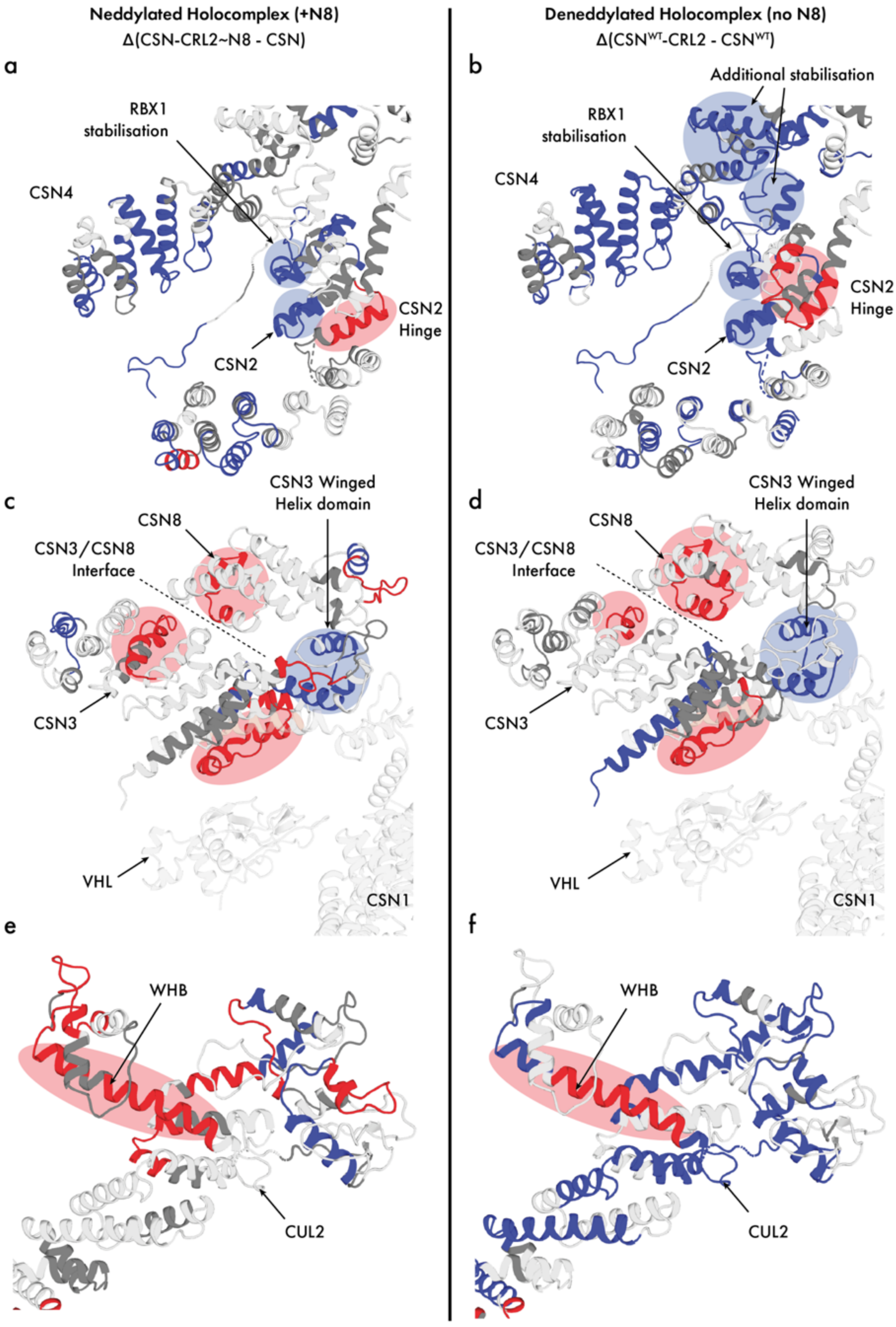
Neddylated Holocomplex (+N8)
Δ(CSN-CRL2~N8 - CSN)
Deneddylated Holocomplex (no N8)
Δ(CSN^WT-CRL2 - CSN^WT)
a
RBX1
stabilisation
CSN4
CSN2
Hinge
CSN2
b
Additional stabilisation
RBX1
stabilisation
CSN4
CSN2
Hinge
CSN2
c
CSN3 Winged
Helix domain
CSN8
CSN3/CSN8
Interface
CSN3
VHL
CSN1
d
CSN3 Winged
Helix domain
CSN8
CSN3/CSN8
Interface
CSN3
VHL
CSN1
e
WHB
CUL2
f
WHB
CUL2

**Supplementary Figure 21. ΔHDX changes in CSN2/CSN4/RBX1, CSN3/CSN8 and CUL2.** Regions showing significant destabilisation (red) and stabilisation (blue) are shown on the structures of CSN-CRL2~N8 (left) and CSN-CRL2 (right) and highlighted for clarity. (**a-b**) Stabilisation of CSN2 and RBX1 interfaces. In the deneddylated complex, CSN4 and RBX1 stabilisation suggests an interface between the two subunits (**b**; top right blue circles). (**c-d**) HDX changes in CSN3, CSN8. VHL and CSN1 (shown in white) are displayed for reference. Both CSN3 and CSN8 experience destabilisation at their interfaces in both left and right complexes. The CSN3 surface closest to VHL (red) and the CSN3 winged helix domain (blue) also shows consistent differences. (**e-f**) Destabilisation of the WHB domain Cullin-2 (CUL2) in both conditions.

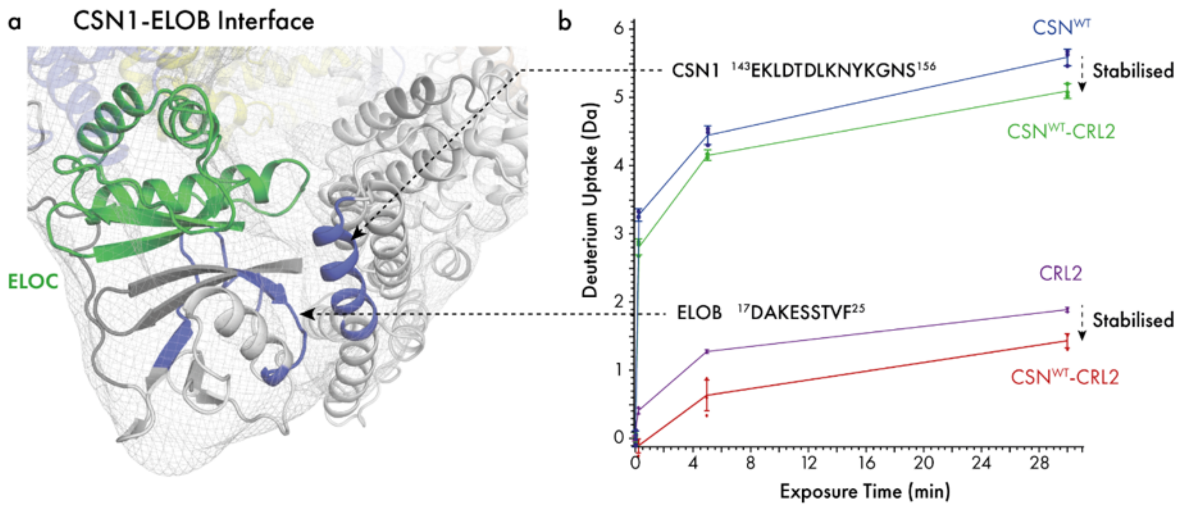


**Supplementary Figure 22. CSN1-ELOB interface of CSN$^{WT}$-CRL2 in HDX-MS.** (a) Structure of CSN-CRL2 fitted to cryo-EM density. Significantly stabilised peptides from CSN1 and ELOB between its interface is highlighted in blue. (b) Deuterium uptake curves over 30 minutes for CSN1 and ELOB peptides are shown for the Δ(CSN$^{WT}$-CRL2 – CSN$^{WT}$) comparison. Error bars represent the deuterium uptake standard deviation. Source Data are provided as a Source Data file.

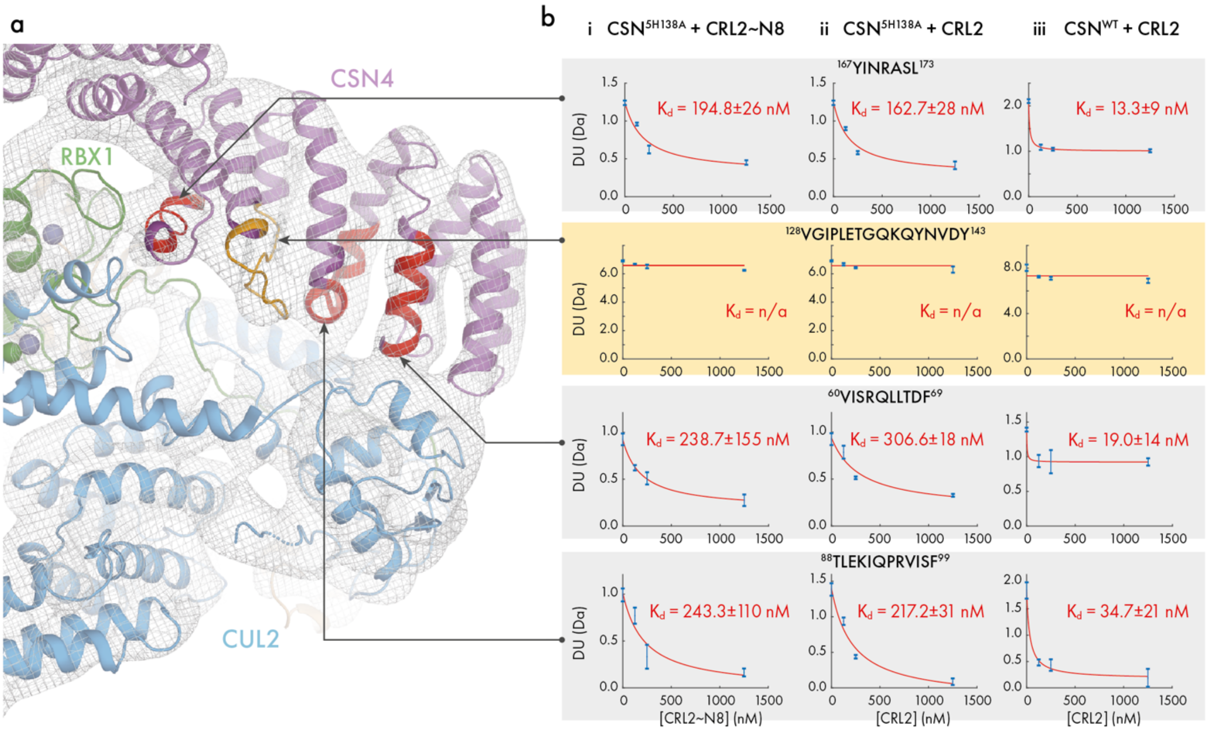


**Supplementary Figure 23. Dissociation constants (kd) between CSN4 and CRL2/CRL2~N8 in CSN-CRL2 complexes.** (a) Structure and density map of the intact CSN-CRL2~N8 shown for reference. Three peptides identified as interacting with CRL2/CRL2~N8 in PLIMSTEX experiments have been highlighted in red. An example of a peptide with no observed changes is shown in orange. (b) PLIMSTEX curve plots showing the deuterium uptake of CSN peptides from (i) CSN-CRL2~N8, (ii) CSN-CRL2 and (iii) $CSN^{WT}$-CRL2, as a function of increasing concentrations of either CRL2 or CRL2~N8. Data points represent the average deuterium uptake and error bars indicate standard deviation of technical triplicates. The red curve for interacting peptides was fitted using a 3-parameter 1:1 binding model for 250 nM CSN or $CSN^{WT}$, titrated with CRL2 or CRL2~N8 from 1:0 to 1:5 molar ratios. Kd ± values denote the standard deviation of the Kd measurement from technical triplicates. Source Data are provided as a Source Data file.

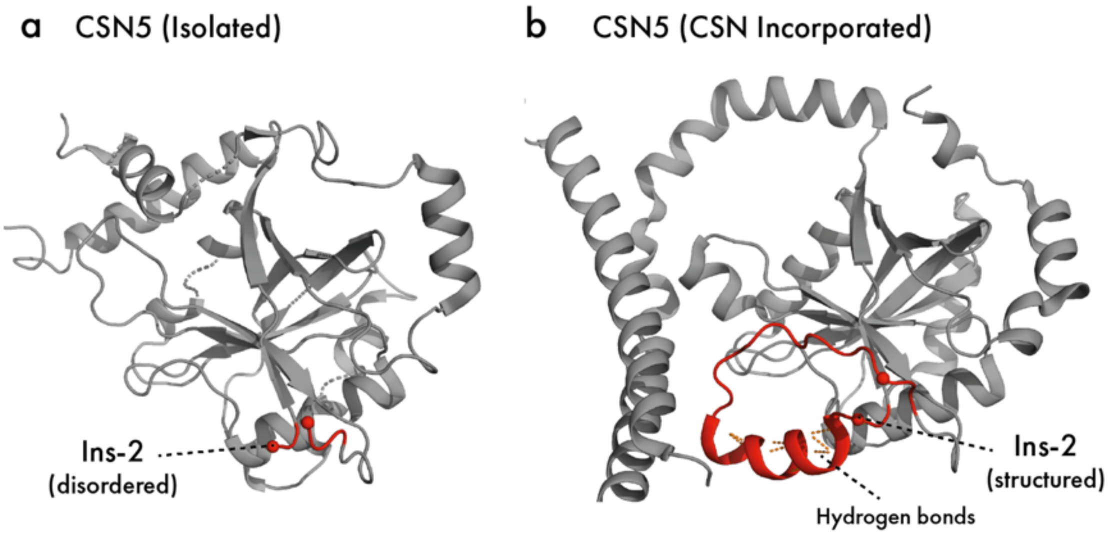


**Supplementary Figure 24. CSN5 Ins-2 loop in isolated and CSN incorporated structures.** (**a**) Disordered Ins-2 loop in isolated CSN (PDB 4F7O). (**b**) Structured Ins-2 loop in CSN incorporated CSN5 (PDB 4D10). Hydrogen bonds of the Ins-2 helix have been shown in orange.

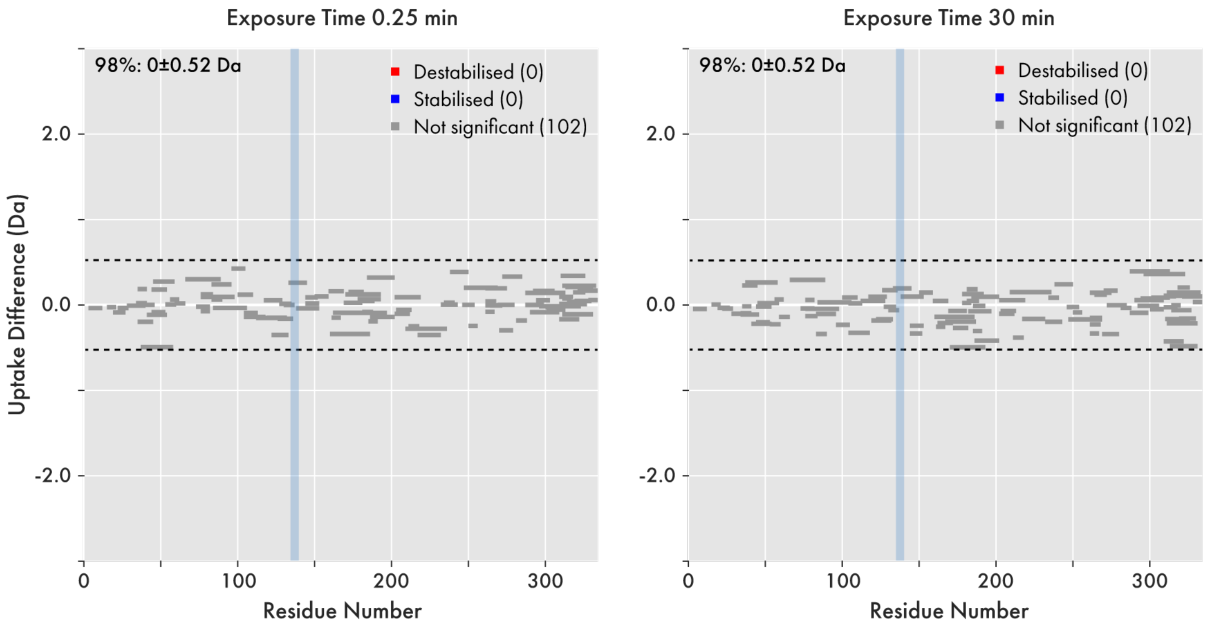


**Supplementary Figure 25. Woods plot comparing deuterium uptake difference of CSN5 peptides from apo-CSN$^{WT}$ and CSN$^{5H138A}$ complexes.** Each CSN5 peptide is represented by a horizontal bar and are coloured according to the significance of its uptake difference. Dotted line represents a 98% confidence interval used to filter peptides for statistical significance. All 102 peptides identified from CSN5 showed non-statistically significant changes in deuterium uptake. The position of the CSN5 H138A mutation has been highlighted by the blue box. Plots generated using Deuteros (v1.08). Details of data can be found in Supplementary Data 1.

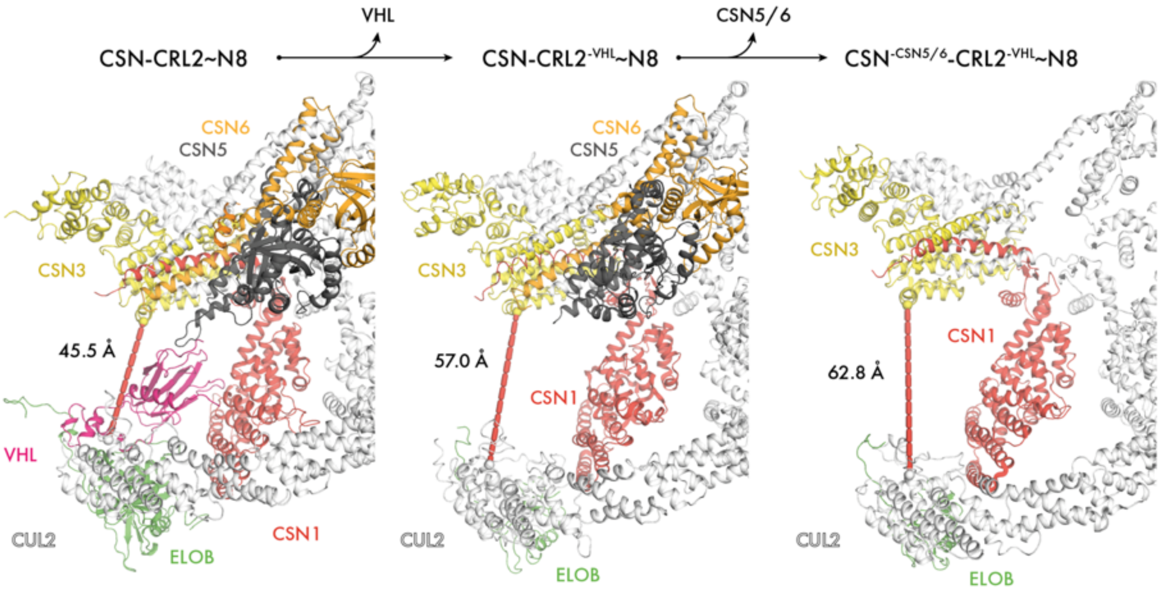


**Supplementary Figure 26. Conformational heterogeneity of the CSN-CRL2~N8 structures.** The distance between CSN3-CUL2 N-terminal domain of CSN-CRL2~N8, after loss of VHL and after loss of CSN5/CSN6 is shown by the dashed red line. All other CSN-CRL2 subunits have been coloured in white for clarity.

# Supplementary Tables

**Supplementary Table 1. Kd values determined for CSN-CRL1[2] and CSN-CRL2 complexes (average from PLIMSTEX)**

| | Kd (nM) | | | |
|---|---|---|---|---|
| | CRL1 | CRL1~N8 | CRL2 | CRL2~N8 |
| $CSN^{WT}$ | 310.0 | - | 22.3 | - |
| $CSN^{5H138A}$ | 10.0 | 1.6 | 228.8 | 225.6 |

**Supplementary Note 1. Multi-template homology modelling of CRL2 using MODELLER**

```
from modeller import *
from modeller.automodel import *

log.verbose()    # request verbose output
env = environ(rand_seed=-556)

# Read in HETATM records from template PDBs
env.io.hetatm = True

a = automodel(env, alnfile='alignment.ali',

knowns=('5N4W_noWHA','4WQO','CRL2_1LDJ_model'),sequence='CRL2',assess_m
ethods=(assess.DOPE, assess.GA341))
a.initial_malign3d = False
a.starting_model = 1
a.ending_model = 1
a.md_level = refine.slow
a.make()

# Run script using 'mod9.16 model_mult.py'
```

**Supplementary Note 2. IMP XL-modelling script for positioning of subunits**

```
import IMP
import IMP.core
import IMP.algebra
import IMP.atom
import IMP.container

import IMP.pmi.restraints.crosslinking
import IMP.pmi.restraints.stereochemistry
import IMP.pmi.restraints.em
import IMP.pmi.restraints.basic
import IMP.pmi.representation
import IMP.pmi.tools
import IMP.pmi.samplers
import IMP.pmi.output
import IMP.pmi.macros
import IMP.pmi.topology

import os
import sys
import csv

# Define Input Files
datadirectory = "./inputs/"
topology_file = datadirectory+"topology.txt"

# Set MC Sampling Parameters
num_frames = 1000
if '--test' in sys.argv: num_frames=50
num_mc_steps = 10
rb_max_trans = 2.00
rb_max_rot = 0.1
bead_max_trans = 0.05

rigid_bodies = [["CUL2-WHB"],["VHL"],['NEDD8']]

# Build the Model Representation
m = IMP.Model()
```

```
# Create list of components from topology file
topology = IMP.pmi.topology.TopologyReader(topology_file)
domains = topology.component_list

print('#'*10,domains)

bm = IMP.pmi.macros.BuildModel(m,
                    component_topologies=domains,
                    list_of_rigid_bodies=rigid_bodies)
representation = bm.get_representation()

# add colors to the components
for nc,component in enumerate(domains):
    name = component.name
    sel = IMP.atom.Selection(representation.prot,molecule=name)
    ps = sel.get_selected_particles()
    clr = IMP.display.get_rgb_color(float(nc)/len(domains))
    for p in ps:
        if not IMP.display.Colored.get_is_setup(p):
            IMP.display.Colored.setup_particle(p,clr)
        else:
            IMP.display.Colored(p).set_color(clr)

# Define Degrees of Freedom
representation.set_rigid_bodies_max_rot(rb_max_rot)
representation.set_floppy_bodies_max_trans(bead_max_trans)
representation.set_rigid_bodies_max_trans(rb_max_trans)

outputobjects = []
sampleobjects = []

outputobjects.append(representation)
sampleobjects.append(representation)

# Excluded Volume Restraint
ev = IMP.pmi.restraints.stereochemistry.ExcludedVolumeSphere(
                                            representation, resolution=10)
ev.add_to_model()
outputobjects.append(ev)
```

```
# Crosslinks - dataset 1
columnmap={}
columnmap["Protein1"]="prot1"
columnmap["Protein2"]="prot2"
columnmap["Residue1"]="res1"
columnmap["Residue2"]="res2"
columnmap["IDScore"]=None

# Experimentally measured crosslinks
xl1 = IMP.pmi.restraints.crosslinking.ISDCrossLinkMS(representation,
                                   datadirectory+'xlinks_exp.txt',
                                   length=35.0,
                                   slope=0.1,
                                   columnmapping=columnmap,
                                   resolution=1.0,
                                   label="Inter-crosslinks",
                                   csvfile=True)
xl1.add_to_model()
sampleobjects.append(xl1)
outputobjects.append(xl1)

# Pseudocovalent crosslinks for keeping complex integrity and allowing
flexibility of long flexible domains
xl2 = IMP.pmi.restraints.crosslinking.ISDCrossLinkMS(representation,

datadirectory+'xlinks_rigidbody_floppy_pseudocovalent.txt',
                                   length=5.0,
                                   slope=1,
                                   columnmapping=columnmap,
                                   resolution=1.0,
                                   label="primary_covalents",
                                   csvfile=True)
xl2.add_to_model()
sampleobjects.append(xl2)
outputobjects.append(xl2)

# Pseudocovalent crosslinks for keeping complex integrity and allowing
flexibility of long flexible domains
xl3 = IMP.pmi.restraints.crosslinking.ISDCrossLinkMS(representation,
```

```
datadirectory+'xlinks_rigidbody_floppy_isopeptide.txt',
                                   length=3.0,
                                   slope=1,
                                   columnmapping=columnmap,
                                   resolution=1.0,
                                   label="primary_covalents",
                                   csvfile=True)
xl3.add_to_model()
sampleobjects.append(xl3)
outputobjects.append(xl3)

mc1=IMP.pmi.macros.ReplicaExchange0(m,
                                    representation,

monte_carlo_sample_objects=sampleobjects,
                                    output_objects=outputobjects,
                                    monte_carlo_temperature=1.0,
                                    crosslink_restraints=[xl1,xl2,xl3],
                                    simulated_annealing=False,
                                    number_of_best_scoring_models=100,
                                    monte_carlo_steps=num_mc_steps,
                                    number_of_frames=num_frames,
                                    global_output_directory="output",
                                    atomistic=True)

mc1.execute_macro() # start

# Run script using 'python CSN_XL_modeling.py'
```

## Supplementary References


1 Scheres, S. H. RELION: implementation of a Bayesian approach to cryo-EM structure determination. *J Struct Biol* **180**, 519-530, doi:10.1016/j.jsb.2012.09.006 (2012).

2 Mosadeghi, R. *et al.* Structural and kinetic analysis of the COP9-Signalosome activation and the cullin-RING ubiquitin ligase deneddylation cycle. *Elife* **5**, doi:10.7554/eLife.12102 (2016).
