# Supplementary material for: Structural basis of Cullin 2 RING E3 ligase regulation by the COP9 signalosome
Source: Nat Commun. 2019 Aug 23;10:3814. doi: 10.1038/s41467-019-11772-y (PMC6707232; doi:10.1038/s41467-019-11772-y)
Supplement: Supplementary file 3 — Description of Additional Supplementary Files [file 41467_2019_11772_MOESM3_ESM.docx]

**Description of Supplementary Files**

**File Name: Supplementary Data 1**

**Description:** Summary file for all data for CSN-CRL2 complexes. Includes per-technique raw data file lists and deposition information for each CSN-CRL2 complex.

**File Name: Supplementary Data 2**

**Description:** Chemical cross-links of the CSN-CRL2~N8 complex.

**File Name: Supplementary Data 3**

**Description:** Chemical cross-links of the CSN-CRL2 complex.

**File Name: Supplementary Data 4**

**Description:** Chemical cross-links of the CSN complex.

**File Name:** **Supplementary Movie 1**

**Description:** Structural basis of Cullin 2 RING E3 ligase regulation by the COP9 signalosome.
