# Supplementary material for: Structural basis of Cullin 2 RING E3 ligase regulation by the COP9 signalosome
Source: Nat Commun. 2019 Aug 23;10:3814. doi: 10.1038/s41467-019-11772-y (PMC6707232; doi:10.1038/s41467-019-11772-y)
Supplement: Supplementary file 9 — Reporting Summary [file 41467_2019_11772_MOESM9_ESM.pdf]

nature research

Corresponding author(s): Argyris Politis, Edward P. Morris

Reporting Summary

Nature Research wishes to improve the reproducibility of the work that we publish. This form provides structure for consistency and transparency in reporting. For further information on Nature Research policies, see Authors & Referees and the Editorial Policy Checklist.

Statistical parameters

When statistical analyses are reported, confirm that the following items are present in the relevant location (e.g. figure legend, table legend, main text, or Methods section).

n/a	Confirmed	
☐	☒	The exact sample size (*n*) for each experimental group/condition, given as a discrete number and unit of measurement
☐	☒	An indication of whether measurements were taken from distinct samples or whether the same sample was measured repeatedly
☐	☒	The statistical test(s) used AND whether they are one- or two-sided<br>*Only common tests should be described solely by name; describe more complex techniques in the Methods section.*
☐	☒	A description of all covariates tested
☒	☐	A description of any assumptions or corrections, such as tests of normality and adjustment for multiple comparisons
☐	☒	A full description of the statistics including central tendency (e.g. means) or other basic estimates (e.g. regression coefficient) AND variation (e.g. standard deviation) or associated estimates of uncertainty (e.g. confidence intervals)
☐	☒	For null hypothesis testing, the test statistic (e.g. *F*, *t*, *r*) with confidence intervals, effect sizes, degrees of freedom and *P* value noted<br>*Give P values as exact values whenever suitable.*
☒	☐	For Bayesian analysis, information on the choice of priors and Markov chain Monte Carlo settings
☒	☐	For hierarchical and complex designs, identification of the appropriate level for tests and full reporting of outcomes
☒	☐	Estimates of effect sizes (e.g. Cohen's *d*, Pearson's *r*), indicating how they were calculated
☐	☒	Clearly defined error bars<br>*State explicitly what error bars represent (e.g. SD, SE, CI)*

Our web collection on statistics for biologists may be useful.

Software and code

Policy information about availability of computer code

Data collection	All software used for data collection detailed in manuscript.
Data analysis	All data analysis methods are detailed in the manuscript.

For manuscripts utilizing custom algorithms or software that are central to the research but not yet described in published literature, software must be made available to editors/reviewers upon request. We strongly encourage code deposition in a community repository (e.g. GitHub). See the Nature Research guidelines for submitting code & software for further information.

Data

Policy information about availability of data

All manuscripts must include a data availability statement. This statement should provide the following information, where applicable:

- Accession codes, unique identifiers, or web links for publicly available datasets
- A list of figures that have associated raw data
- A description of any restrictions on data availability

The cryo-EM density maps described here have been deposited in the Electron Microscopy Data Bank (EMDB) under EMD-4739 [https://www.ebi.ac.uk/pdbe/entry/emdb/EMD-4739], EMD-4744 [https://www.ebi.ac.uk/pdbe/entry/emdb/EMD-4744], EMD-4742 [https://www.ebi.ac.uk/pdbe/entry/emdb/EMD-4742], EMD-4736 [https://www.ebi.ac.uk/pdbe/entry/emdb/EMD-4736] and EMD-4741 [https://www.ebi.ac.uk/pdbe/entry/emdb/EMD-4741]. All of the model coordinate sets fitted

April 2018

to the cryo-EM maps have been deposited in the Protein Data Bank (PDB) under 6R7F [http://dx.doi.org/10.2210/pdb6R7F/pdb], 6R7N [http://dx.doi.org/10.2210/pdb6R7N/pdb], 6R7I [http://dx.doi.org/10.2210/pdb6R7I/pdb], 6R6H [http://dx.doi.org/10.2210/pdb6R6H/pdb] and 6R7H [http://dx.doi.org/10.2210/pdb6R7H/pdb]. All XL-MS, HDX-MS and native MS data were deposited to the proteomeXchange repository under accession: PXD013001 and PXD013018. A list of data collected for each technique and complex is available in the Supplementary Data file. The source data for Figures 3, 4 and Supplementary Figures 2, 3, 5, 8, 9, 10, 11, 12, 16, 17, 18, 20, 21 and 23 are detailed in the Source Data file.

Field-specific reporting

Please select the best fit for your research. If you are not sure, read the appropriate sections before making your selection.

☒ Life sciences ☐ Behavioural & social sciences ☐ Ecological, evolutionary & environmental sciences

For a reference copy of the document with all sections, see nature.com/authors/policies/ReportingSummary-flat.pdf

Life sciences study design

All studies must disclose on these points even when the disclosure is negative.

Sample size	Does not apply to this study.
Data exclusions	No data were excluded from this study.
Replication	All HDX-MS experiments were performed as technical triplicates.
Randomization	Does not apply to this study.
Blinding	Does not apply to this study.

Reporting for specific materials, systems and methods

Materials & experimental systems

n/a	Involved in the study
☒	☐ Unique biological materials
☒	☐ Antibodies
☐	☒ Eukaryotic cell lines
☒	☐ Palaeontology
☒	☐ Animals and other organisms
☒	☐ Human research participants

Methods

n/a	Involved in the study
☒	☐ ChIP-seq
☒	☐ Flow cytometry
☒	☐ MRI-based neuroimaging

Eukaryotic cell lines

Policy information about cell lines

Cell line source(s)	High Five Cells (BTI-TN-5B1-4, from embryonic tissue of the cabbage looper, Trichoplusia ni, Life Technologies #B85502)
Authentication	None of the cell lines used were authenticated.
Mycoplasma contamination	Cell lines were not tested for mycoplasma contamination.
Commonly misidentified lines (See ICLAC register)	None
